# Type II porous ionic liquid based on metal-organic cages that enables l-tryptophan identification

**DOI:** 10.1038/s41467-022-30092-2

**Published:** 2022-04-29

**Authors:** Zhuxiu Zhang, Baolin Yang, Bingjie Zhang, Mifen Cui, Jihai Tang, Xu Qiao

**Affiliations:** 1 State Key Laboratory of Materials-Oriented Chemical Engineering, College of Chemical Engineering, Nanjing Tech University, No. 30 Puzhunan Road, 211816 Nanjing, China; 2 Jiangsu National Synergetic Innovation Centre for Advanced Materials (SICAM), No. 5 Xinmofan Road, 210009 Nanjing, China

**Keywords:** Organic-inorganic nanostructures, Molecular capsules, Metal-organic frameworks, Soft materials

## Abstract

Porous liquids with chemical separation properties are quite well-studied in general, but there is only a handful of reports in the context of identification and separation of non-gaseous molecules. Herein, we report a Type II porous ionic liquid composed of coordination cages that exhibits exceptional selectivity towards l-tryptophan (l-Trp) over other aromatic amino acids. A previously known class of anionic organic–inorganic hybrid doughnut-like cage (HD) is dissolved in trihexyltetradecylphosphonium chloride (THTP_Cl). The resulting liquid, HD/THTP_Cl, is thereby composed of common components, facile to prepare, and exhibit room temperature fluidity. The permanent porosity is manifested by the high-pressure isotherm for CH_4_ and modeling studies. With evidence from time-dependent amino acid uptake, competitive extraction studies and molecular dynamic simulations, HD/THTP_Cl exhibit better selectivity towards l-Trp than other solid state sorbents, and we attribute it to not only the intrinsic porosity of HD but also the host-guest interactions between HD and l-Trp. Specifically, each HD unit is filled with nearly 5 l-Trp molecules, which is higher than the l-Trp occupation in the structure unit of other benchmark metal-organic frameworks.

## Abstract (web summary)

Porous liquids are potentially useful materials for the identification and separation of non-gaseous compounds. Herein, the authors report a type II porous ionic liquid with permanent porosity and high selectivity towards l-tryptophan (l-Trp) over other aromatic amino acids.

## Introduction

Porous liquids represent an emerging class of materials that combine the benefits of porosity and fluid properties in a rational manner^1–3^. The intrinsic porosities of porous liquids are mainly derived from components with either zero dimensional nanostructures (e.g. molecular cages^4^, hollow silica^5^) or extended network structures (e.g. metal-organic frameworks^6^ and zeolites^7^). Their modular nature and structure diversity facilitate the exquisite control over porous liquids with respect to the pore size and chemistry through the judicious selection of structural constituent. Such versatile porosities thereby enable systematic studies that afford an understanding of structure-function relationships of porous liquids, which has led them to be of particular interest for potential utility in gas storage^8–16^, separations^17–20^ and catalysis^21–23^.

More than one third of pore liquids consist of molecular cages including porous organic cages (POCs) and metal-organic cages (MOCs) as pore hosts. Molecular cages are generally solid state materials at atmospheric condition. The functionalization of their periphery with alkyl chains^24,25^ or poly(ethylene glycol) chains^4^ offers opportunities to reduce the melting point, giving rise to the Type I porous liquids. Another synthetic approach of Type I porous liquids is to transform the anionic POC into ionic liquid by pairing with cationic 18-crown-6/K^+^ complex^26^. However, most of molecular cage-derived porous liquids are generated via dissolving molecular cages in bulky solvents, also known as Type II porous liquids. The dissolution process is essentially simpler than multi-step organic synthesis involved in the synthesis of Type I porous liquids. MOCs^27–29^ are an important class of discrete nanoscale structures amenable to crystal engineering^30,31^ and can be rationally designed using the ‘node-and-spacer’ approach. The pre-selection of nodes and spacers enables the fine-tuning of structure and properties, so that MOCs can be regarded as ideal pore hosts for Type II porous liquids. In fact, MOCs have been explored in liquid phase to extract target molecules from another immiscible liquid phase via host-guest binding affinity^32^. There is also a handful of papers regarding the gas encapsulation in cavities of MOCs in solution^33^. However, the use of volatile solvent and the unwanted occupation of the cage cavity fails to endow the solution of MOCs with permanent porosity. To our knowledge, there is only one MOC-based Type II porous liquid generated by dissolving MOP-18 in 15-crown-5^18^. In this contribution, we introduce a class of Type II porous liquids composed of anionic organic–inorganic hybrid doughnut-like nanostructures (HD)^34^ dissolved in trihexyltetradecylphosphonium chloride (THTP_Cl). Note that this is also an underexplored example of Type II porous ionic liquid based upon MOCs.

Molecular cages, including those serve as the “doughnut-shaped” host (e.g. cyclodextrin and cucurbiturils), have been of particular interest in the contemporary supramolecular chemistry^35^. Porous liquids consisting of molecular cages thereby exhibit host-guest properties, as exemplified by the shape and size selectivity for a series of isomeric alcohols in Type I porous ionic liquids composed of tetrahedral coordination cage^4^. Most recently, Xia et al. reported the use of the cyclodextrin-derived Type I porous liquid for the efficient chiral recognition and separation of nucleosides in the solution^36^. Except for the two examples above, current research on the host-guest properties of the molecular cage-based porous liquids has been mainly focused on the accommodation of small gaseous molecules such as carbon dioxide^8,37,38^, methane^11,14,39^ and chlorofluorocarbon^4^. It still remains a rare and poorly understood the capture of non-gaseous guest molecules in porous liquids. The downstream processing of naturally derived amino acids include chromatographic steps for the separation and purification of amino acids. Porous solids such as ion-exchange resin have been used for the chromatographic separation of amino acid, which necessarily entail batch operations that accounts for 80% of the amino acid cost. Hence, there is a critical need to develop alternative selective separation process for amino acids with high purity and low cost.^40^. Against the greatly explored batch or semi-batch processes involving multiple energy-intensive unit operations, the continuous production scheme which promise much higher productivity and higher acid concentration can be considered good alternatives^41^. In this context, the use of the porous liquid as the porous extraction medium, which has the potential to be incorporated in the continuous flow process^42^, is likely to generate versatile platforms for the isolation and purification of amino acid from the fermentation broth.

In this work, we report the use of the Type II porous ionic liquid (HD/THTP_Cl) based upon anionic organic–inorganic hybrid doughnut-like cage for the selective extraction of l-tryptophan (l-Trp) from the aromatic amino acid mixtures in aqueous solution. HD/THTP_Cl is characterized by several techniques covering the key properties including the thermal properties, rheological properties, permanent porosity and viscosity. HD/THTP_Cl exhibits not only higher l-Trp selectivity than other typical porous liquids but also larger unit occupation of l-Trp than other benchmark porous coordination polymers. We attribute such exceptional performance to the intrinsic porosity of HD and exceptional l-Trp binding affinity as revealed by modeling studies. The synthetic approach of HD/THTP_Cl can be extended to a broad family of metal-organic cages. The use of HD/THTP_Cl as the task-specific extractant represent a broad paradigm to target the selectivity towards other biomolecules as exemplified by the selective extraction of d-ribose from the aqueous solution of d-ribose and d-glucose.

## Result and discussion

### Synthesis and purification

**Fig. 1:**
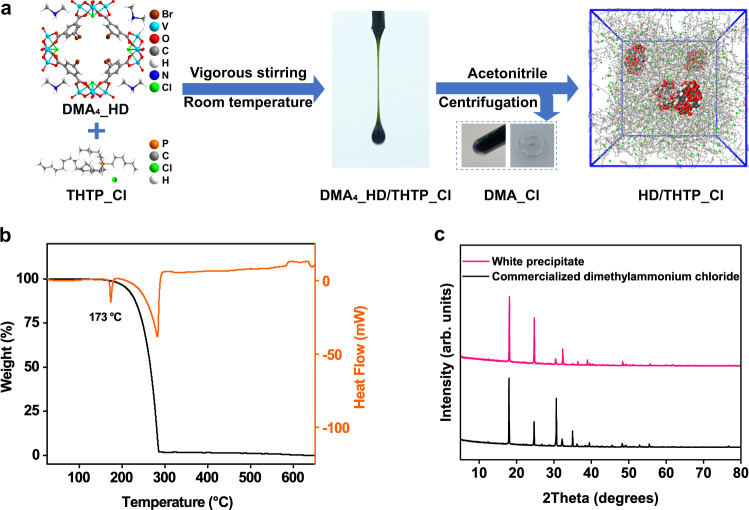
Synthesis of HD/THTP_Cl and characterization of the removed white precipitate characterization. **a** Schematic illustration of the synthesis of HD/THTP_Cl. **b** TG-DSC curve of the white precipitate. **c** Experimental PXRD pattern of the white precipitate and commercialized dimethylammonium chloride.

The crystallization of anionic organic–inorganic hybrid nanodoughnut (HD) with dimethylammonium (DMA) as the counter-ion occurs during the solvothermal reaction of 5-bromo-1,3-benzenedicarboxylatic acid and VCl_3_ in DMF. The resulting DMA_4__HD crystals are difficult to be dissolved in organic solvents (e.g. 15-crown ether and hexachloropropene) that have been used for the synthesis of Type II porous liquids, because DMA_4__HD is essentially a type of salt difficult to be ionic dissociated in the aprotic organic solvent. Attempts dissolve DMA_4__HD in imidazole/pyridine/ammonium-based ionic liquids were unsuccessful. Fortunately, we observed the readily dissolution of DMA_4__HD in trihexyltetradecylphosphonium chloride (THTP_Cl) to form the dark green liquid (DMA_4__HD/THTP_Cl) via stirring at room temperature (Fig. 1a). THTP_Cl is a branched ionic liquid of which the branched topology and molecular size can prevent itself from entering the nano-sized pores^43^. It’s necessary to remove DMA from DMA_4__HD/THTP_Cl because it may occupy the cavity of HD in the solution. The addition of acetonitrile to DMA_4__HD/THTP_Cl formed a certain amount of the white precipitate with the melting point (*T*_m_) of 173 °C corresponding to the pure dimethylammonium chloride (Fig. 1b). The bulk purity of the precipitate was furtherly verified by comparing its X-ray powder diffraction pattern with commercialized dimethylammonium chloride (Fig. 1c). The content of C, H and N in precipitate was respectively verified as 28.83%, 9.32% and 17.03% via elemental analysis (Calculated: C 29.45%; H 9.88%; N 17.17%). The presence of methyl group in the white precipitate was determined by ^1^H NMR analysis (Supplementary Fig. 1). Thus, the white precipitate can be verified as the pure dimethyammonium chloride. The molar ratio of DMA and HD in DMA_4__HD/THTP_Cl was calculated through the moles of white precipitate divided by the moles HD in HD/THTP_Cl. Considering the inevitable small amount of weight loss during the purification, we think the resulting molar ratio of 3.85:1 is consistent with the molecular formula of as-synthesized DMA_4__HD. To be specific, the dark green liquid (DMA_4__HD/THTP_Cl, 5.55 g) contains 0.1 mmol of DMA_4__HD and 10 mmol of THTP_Cl. It thereby needs 0.4 mmol of Cl anions from THTP_Cl to form dimethylammonium (DMA_Cl). Once the DMA_Cl was removed by adding acetonitrile, the remaining liquid contains 0.1 mmol of HD^4−^ anions, 9.6 mmol of Cl^−^ anion and 10 mmol of THTP^+^ cation. The charge of the liquid was thereby balanced.

### Structural characterization

**Fig. 2:**
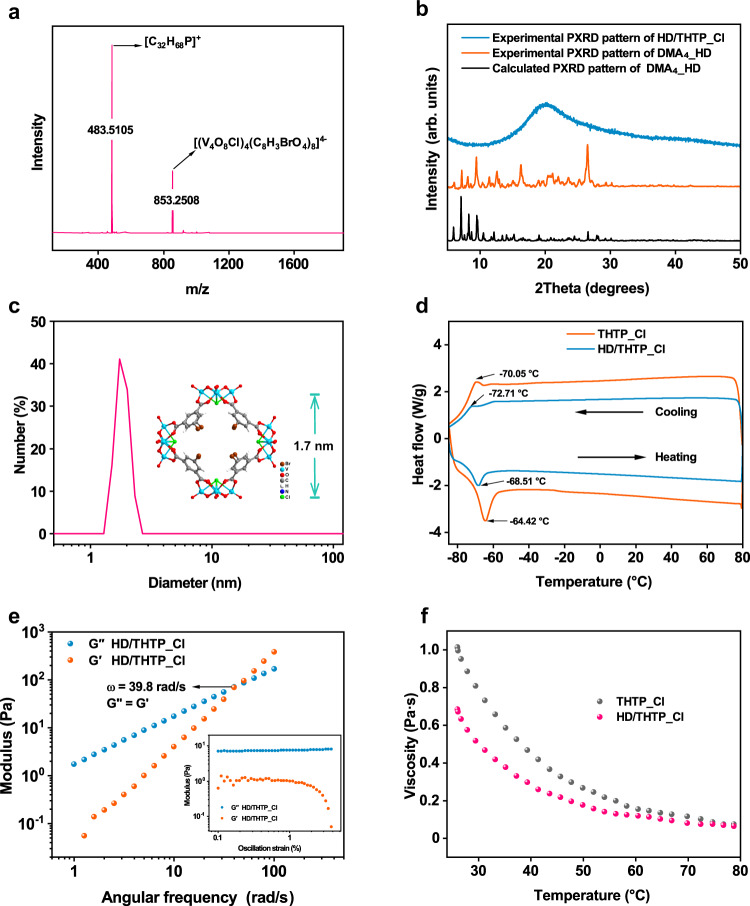
Structure characterizations of HD/THTP_Cl. **a** QTOF mass spectrum of HD/THTP_Cl. **b** Experimental PXRD patterns of DMA_4__HD and HD/THTP_Cl, and calculated PXRD pattern of DMA_4__HD. **c** Particle size and distribution from DLS measurements of HD/THTP_Cl in acetonitrile. Inset: Molecular structure of HD anion with the diameter of 1.7 nm. **d** DSC cooling and successive heating curves of THTP_Cl and HD/THTP_Cl. **e** Frequency-dependent modulus plots of HD/THTP_Cl. Inset: Strain-dependent modulus plots of HD/THTP_Cl with the angular frequency fixed at 5 rad/s. **f** Viscosity-temperature curves of THTP_Cl and HD/THTP_Cl.

The quadrupole time-of-flight (QTOF) mass spectrum of the HD/THTP_Cl in acetonitrile confirms the existence of HD anion, according to the peaks at the *m/z* of 853.2508 assigned to the molecular formula of [(V_4_O_8_Cl)_4_(C_8_H_3_BrO_4_)_8_]^4−^. Another intense molecular ion peaks at the *m/z* of 483.5105 are assigned to THTP cation, [C_32_H_68_P]^+^ (Fig. 2a). There are no powder X-ray diffraction peaks of HD/THTP_Cl compared to as-synthesized DMA_4__HD, excluding the existence of small HD crystallites dispersed in the liquid (Fig. 2b). All HD anions are fully dissolved in the THTP_Cl as indicated by dynamic light scattering measurements with an average size of 1.8 nm, which is in good agreement with diameter of HD cage (Fig. 2c). Thermogravimetric analysis reveals a similar thermal stability between HD/THTP_Cl and THTP_Cl before decomposition (Supplementary Fig. 2). Differential scanning calorimetry (DSC) curves showed an endothermic/exothermic peaks of HD/THTP_Cl and THTP_Cl of −64.42/−70.05 and −68.51/−72.71 °C respectively, corresponding to the melting temperature (*T*_m_) and crystallization temperature (*T*_c_). HD/THTP_Cl and THTP_Cl exhibited a similar thermal hysteresis behavior related to the reversible first-order structural phase transition^44^. Both *T*_m_ and *T*_c_ of HD/THTP_Cl are slightly higher than those in THTP_Cl, which can be tentatively attributed to inhibition of both chains mobility and vibration of THTP cations by HD anions^45^ (Fig. 2d).

The rheological properties of HD/THTP_Cl were studied by a rotational rheometer. The frequency sweeps were conducted within a linear viscoelastic region between 0.1% and 1% from an initial amplitude sweep (Fig. 2e inset). The liquid-like behavior is reflected by higher loss modulus (G″) than storage modulus (G′) at low angular frequency range. The equivalent modulus value indicates the solid–liquid transition of HD/THTP_Cl at an angular frequency of 39.8 rad/s (Fig. 2e). HD/THTP_Cl shows the evidence of Newtonian fluid as the linear dependence of shear stress on the shear rate (Supplementary Fig. 3). The viscosity of HD/THTP_Cl thereby remain invariant of 0.7 Pa·s with increasing shear rate (Supplementary Fig. 4), and it was even lower than the viscosity of pure THTP_Cl at 25 °C (1 Pa·s) (Fig. 2f).

### Demonstration of porosity in HD/THTP_Cl

**Fig. 3:**
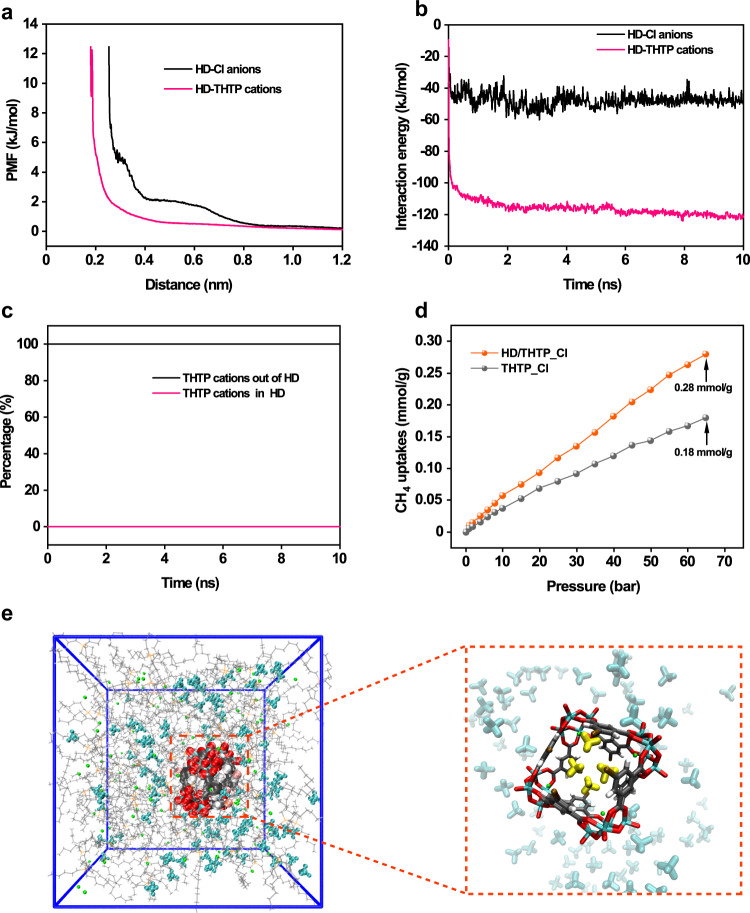
Porosity measurements. **a** Center-of-mass—a-particular-atom PMF for HD –THTP cations (P atom) and HD–Cl anions (Cl atom) from MD simulations. **b** The interaction energy of HD anion with THTP cation and chloride anion. **c** Simulated distribution percentage of THTP cation in and out of the cavity of HD. **d** High-pressure CH_4_ absorption data of HD/THTP_Cl and THTP_Cl. **e** The simulation box of methane in HD/THTP_Cl at 25 °C and 65 bar, in which the HD anion is occupied by four methane molecules at 10 ns. (Methane molecule inside and outside of HD are respectively highlighted with yellow and cyan).

Molecular Dynamics (MD) simulations of a bulk system consisting of 5 HD anions, 500 THTP cations and 480 chloride anions in a cubic and periodic simulation box were carried out in the NPT ensemble at 1 bar and 298 K with a time-step of 2 fs. To estimate the energy barrier for THTP cations and chloride anions transporting through the HD anion, the potential of mean force (PMF) was studied via radial distribution functions. The approaching of THTP cations and chloride anions to HD causes the significant increase of PMF from 0 to 12.5 kJ/mol, so that the minimum distance between HD and chloride anions are at approximately 0.8 nm (Fig. 3a). In contrast, the distance between HD anion and THTP cation can be as close as approximately 0.45 nm, which is consistent with the larger interaction energy of 120 kJ/mol than that of 50 kJ/mol between HD anion and chloride anion due to electrostatic repulsion (Fig. 3b). On average, around 100% of the THTP cations at any given time are outside the HD anions so that there is enough void cavities in HD/THTP_Cl (Fig. 3c).

The MD simulation result detailed herein prompted us to evaluate the high-pressure CH_4_ absorption ability of HD/THTP_Cl from 0 to 65 bar, and THTP_Cl was also chosen as the contrast. The gravimetric CH_4_ uptakes (298 K, 65 bar) in HD/THTP_Cl and THTP_Cl are 0.28 and 0.18 mmol/g respectively (Fig. 3d). We then carried out a simulation of the 1:100:96 mixture of HD, THTP and chloride ions combined with 100 methane molecules at 1, 5 and 10 bars, respectively. The starting configuration was generated by randomly placing 100 methane molecules in the bulk fluid without any atomic overlaps. The system was then relaxed for 10 ns under NPT conditions, and all methane molecules were observed freely flowed in HD/THTP_Cl during the simulations. Supplementary Fig. 5 shows the number of methane molecules residing in the HD cavity as a function of time during the production run. The average number of methane molecules residing in HD cavity derived from MD simulation and the number of methane molecules encapsulated by HD cavity calculated from high-pressure methane absorption data were listed in Supplementary Table 1. It is interesting to find that simulated number of methane molecules is higher than the experimental ones at pressure from 1 to 10 bar. The higher the pressure, the closer the experimental number of methane molecules towards the simulated number. In this case, we tentatively attribute this phenomenon to the limited mass transfer of methane molecules in the viscous porous liquid. The viscosity of HD/THTP_Cl was 0.7 Pa·s at 25 °C, which was nearly 700 times larger than the viscosity of water. Thus, the mass transfer of methane molecules in HD/THTP_Cl at low pressure is supposed to be reduced^46^. In contrast, the high pressure is likely to provide stronger driving force for the methane molecules to move into the HD cavity^47^. This is the reason why the simulated and experimental number of methane molecules are quite close at 65 bar. There are on average 4.72 methane molecules residing the HD cavity at 65 bar according to the MD simulations, which means the number of methane molecules in the HD cavity kept fluctuating during simulation process. Figure 3e presents the simulation systems at the running time of 10 ns, where there are exactly 4 methane molecules encapsulated in the HD cavity. According to Supplementary Fig. 5d, other running times around 10 ns may exhibit the simulation system containing 3 or 5 methane molecules encapsulated in the HD cavity.

### Selective extraction of l_-_tryptophan from aromatic amino acids mixture

**Fig. 4:**
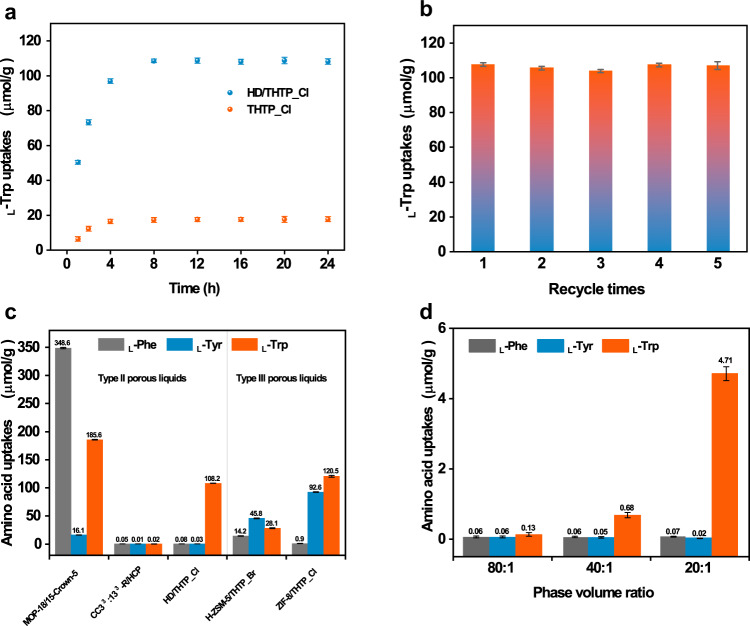
Selective extraction of l-Trp. **a** l-Trp uptake over time in HD/THTP_Cl and THTP_Cl. **b** The reuse of HD/THTP_Cl for the single-compound extraction of l-Trp. **c** The uptake of amino acids in MOP-18/15-crown-5, CC3^3^:13^3^-R/HCP, HD/THTP_Cl, H-ZSM-5/THTP_Br and ZIF-8/THTP_Cl. **d** Effect of the phase volume ratio on the l-Trp selectivity.

l-Tryptophan (l-Trp) is one of the eight essential amino acids that play a key role in human metabolism as exemplified by the biosynthesis of neurotransmitters, hormones, and vitamins. The isolation and purification of l-Trp from the fermentation broth that contains l-tyrosine (l-Tyr) and l-phenylalanine (l-Phe) as major impurities is an important for the l-Trp production. Liquid−liquid extraction is one of the widely used separation methods, and experiments were carried out by allowing 0.1 mL of HD/THTP_Cl come into contact with 2 mL of aqueous solution of amino acid. The concentration of l-Trp, l-Tyr and l-Phe is 0.05 mol/L, 0.15 mol/L and 0.002 mol/L respectively. All three concentrations are close to the maximum concentrations of corresponding amino acids^48^. For the single-component system, Fig. 4a presents a significant increase of the extraction rate before equilibrium as indicated by the incremental concentration of l-Trp in the HD/THTP_Cl phase during the timeframe of 1–4 h. The extraction reached equilibrium at 8 h as exhibited by the plateau from kinetic curves, and the final l-Trp uptake was 108.2 µmol/g after 24 h. Comparably, the extraction ability of THTP_Cl was significantly reduced to 17.7 µmol/g over the whole extraction time range. In addition, neither l-Tyr nor l-Phe was extracted by HD/THTP_Cl or THTP_Cl after 24 h (Supplementary Figs. 6, 7). The reuse of HD/THTP_Cl were realized by adding ethanol/water solution (1:4) for back extraction, followed by being vacuum dried at 60 °C for 2 h. Figure 4b shows that HD/THTP_Cl does not lose its extraction ability after 5 extraction cycles. We have compared the uptake of amino acids in HD/THTP_Cl with other four typical porous liquids in literatures, and none of them exhibited considerable selectivity towards l-Trp. Both of Type III porous liquids use phosphonium-based ionic liquid as the solvent. ZIF-8/THTP_Cl^49^ exhibited similar l-Trp uptake as in HD/THTP_Cl, but the l-Tyr uptake in ZIF-8/THTP_Cl is close to l-Trp. The l-Trp uptake in HZSM-5/THTP_Br^43^ is four times lower than HD/THTP_Cl probably because of its narrow pore size, and the uptake of l-Phe and l-Tyr is also highly reduced too. The CC3^3^:13^3^-R/HCP^14^ is Type II porous liquid based upon porous organic cage dissolved in hexachloropropene, but its ultra-microporosity resulted in the negligible uptake of all three amino acids. MOP-18/15-Crown-5^18^ is another Type II porous liquid based upon metal-organic cage, and it showed considerable uptake of l-Trp (185.6 µmol/g) and l-Phe (348.6 µmol/g) (Fig. 4c and Supplementary Table 3). Therefore, only HD/THTP_Cl exhibited the selectivity towards l-Trp.

To support and confirm the high selectivity derived from the single-component extraction experiments, the selective l-Trp extraction by HD/THTP_Cl from the model solution that contains equimolar mixture of l-Trp, l-Tyr and l-Phe was investigated and presented in Fig. 4d. The initial concentration of each amino acid in water phase is 2 mmol/L with the water: HD/THTP_Cl phase ratio of 20:1, and higher phase ratio means lower initial concentration of amino acids. Both concentrations of l-Tyr and l-Phe are close to zero at phase volume ratio of 80:1, 40:1 and 20:1. The concentration of l-Trp in aqueous phase after extraction is 0.1 mmol/L at the phase volume ratio of 80:1. When the phase volume ratio increase to 20:1, the corresponding concentration of l-Trp in water after extraction is 1.7 mmol/L. The separation factor is 24 for l-Trp to l-Phe and 23 for l-Trp to l-Tyr at the phase ratio of 20:1, whereas the corresponding separation factor are reduced to 0.94 and 0.93 at the phase ratio of 80:1. These results suggest that l-Trp can be selectively separated from a mixture of amino acids by HD/THTP_Cl, and the use of low phase volume ratios, such as 20:1, is beneficial to the high extraction selectivity.

**Fig. 5:**
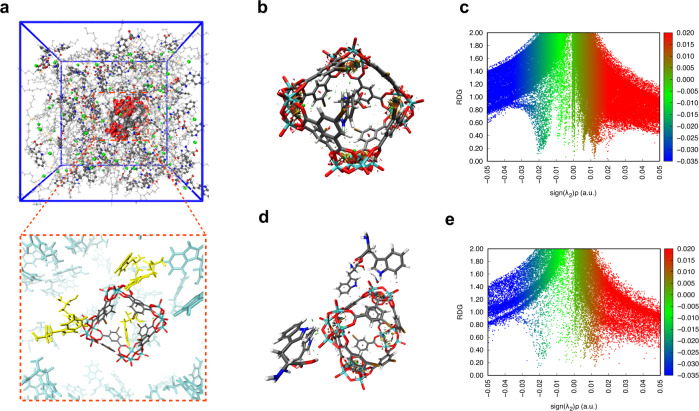
The l-trp recognition ability of HD. **a** The simulation box of l-Trp in HD/THTP_Cl, in which the one molecule resides in the cavity of HD and four molecules interact with polyoxovanadates of HD. (The identified l-Trp molecule are respectively highlighted with yellow.) **b** Color-filled RDG isosurfaces depicting noncovalent interaction regions of the encapsulated-l-Trp and HD cage. **c** Plots of sign (λ_2_)ρ and its reduced density gradient for the encapsulated-l-Trp and HD cage. **d** Color-filled RDG isosurfaces depicting noncovalent interaction regions of the absorption of l-Trp by the periphery of HD cages. **e** Plots of sign (λ_2_)ρ and its reduced density gradient for the absorption of l-Trp by the periphery of HD cages.

The high selectivity toward l-Trp exhibited by HD/THTP_Cl is then addressed through molecular dynamic simulations in which amino acid molecules is added in the simulation box for probing the recognition ability of HD. The simulation system containing l-Trp molecules reach stability within 1 ns and remain stable for the rest simulation time. In comparison, the more fluctuated higher root mean square deviations (RMSD) value of l-Phe and l-Tyr simulation system were found within 2 ns and 3.5 ns, indicating the highly bias and instability of the simulation systems (Supplementary Figs. 8–10 and Supplementary Table 2). There is one l-Trp molecule encapsulated in the cage of HD and four l-Trp molecules surrounding the periphery of HD cage (Fig. 5a). Noncovalent interactions between l-Trp molecule and HD were determined using reduced density gradient (RDG) analysis^50^. The RDG isosurface is colored according to the value of sign(λ_2_)ρ to distinguish the different weak interactions, and the interaction type is illustrated by the blue-green-red scale (blue: strong absorption including hydrogen bond and strong halogen bond, green: van der Waals interactions, red: steric hindrance) (Fig. 5b–e and Supplementary Fig. 11). The van der Waals interaction and C–H···π interactions (*d*[C···centroid_phen_] = 4.2 Å) between the encapsulated l-Trp and HD cage can be clearly detected according to the dispersed low density isosurfaces between l-Trp and the benzene ring of ligand (Supplementary Figs. 12–14). For l-Trp molecules outside the HD cage, the isosurface lies between the nitrogen atom from the indole ring and oxygen atom from the HD cage, indicating several N–H···O interactions with (N···O) distances ranging from 2.4 to 3.4 Å and N–H···O angle ranging from 104° to 158°. RDG analysis also demonstrates that there are several van der Waals interactions between l-Trp and HD cage (Supplementary Figs. 15–18). For MD simulation system containing l-Tyr molecules, there are two l-Tyr molecules surrounding the periphery of HD cage. The N-H···O interaction between amine groups of one l-Tyr and terminal oxygen from HD cage was observed with distance (N···O) of 3.2 Å and N-H···O angle of 153°. Similar interactions were found for another l-Tyr molecule with a distance (N···O) of 3.4 Å and N–H···O angle of 167° (Supplementary Figs. 19–21). For MD simulation system containing l-Phe molecules, there is no observable interatomic contacts among l-Phe molecules and HD cages. The shortest distance between l-Phe and adjacent HD cage is 3.9 Å from nitrogen atoms in l-Phe and terminal oxygen atom in HD, which falls out of the range of hydrogen bond (Supplementary Figs. 22, 23). The structural consequence of weak interaction analysis is that the cavity of HD only exhibited favorable interaction with l-Trp, and N–H···O interaction for the periphery of HD cage for l-Trp is on average stronger than l-Tyr as indicated by the bond distance and bond angle.

Except for the intermolecular interaction, the energy barrier for the amino acid transporting through the HD cage is another factor for the extraction selectivity. The potential of mean force (PMF) was investigated to estimate such energy barrier. The approaching of l-Tyr and l-Phe to HD both cause the significant increase of PMF above 100 kJ/mol, so that the minimum distance between HD and l-Tyr/l-Phe is at approximately 0.3 nm. In contrast, the distance between HD anion and THTP cation can be less than 0.15 nm before the sharp increase of PMF. Moreover, the presence of THTP cation is also important for the exclusion of l-Tyr and l-Phe (Supplementary Fig. 24). We calculated binding energy of amino acids with THTP and HD cage respectively. The binding energy of THTP/l-Trp blend was greater than that of THTP/l-Tyr blend and THTP/l-Phe blend, indicating the stronger compatibility of THTP and l-Trp than that of THTP and l-Tyr or l-Phe. THTP itself is likely to extract more l-Trp molecules than l-Phe and l-Tyr, which is consistent with the experiment result. Importantly, the binding energy of HD/l-Trp blend is ca. 19 kJ/mol larger than THTP/l-Trp blend, whereas the binding energy of HD/l-Phe blend is close to that of THTP/l-Phe (Supplementary Table 4). This result further proved that the presence of HD in THTP_Cl effectively facilitate the affinity of l-Trp for HD rather than l-Phe and l-Tyr.

As with the experimental and modeling results discussed above, l-Trp molecules were not only encapsulated in almost all of the internal cavities of HD/THTP_Cl, but also interacting with the periphery of HD. The number of l-Trp loaded per HD cage unit was calculated to be 4.7. Although the gravimetrical uptake of l-Trp in HD/THTP_Cl is moderate by the standard of porous materials^51^, the unit occupation of HD/THTP_Cl is unprecedented, outperforming other benchmark porous coordination polymers: 1.3 for UIO-66, 0.4 for MIL-140B, 2.1 for MIL-140C, 1.5 for MIL-140D, 2.2 for MOF-808, 2.6 for MIL-68(Al) and 2.7 for zeolite HY calculated via multiplying the gravimetric uptake of l-Trp by the molecular weight contained in one unit cell of certain MOF (Supplementary Fig. 25). The low unit occupation by l-Trp observed in these porous coordination polymers may originate from two factors: (i) limited access of the l-Trp in narrow pore environment. Adsorbents such as UiO-66 have narrow channel (5–11 Å) that are comparable to the l-Trp dimension (11.1 × 6.4 × 8.1 Å) calculated by Multiwfn program^52^, so that the penetration of the l-Trp might be restricted; (ii) restricted diffusion of the l-Trp to the inner structure. The growth of the MOF or zeolite crystalline particle inevitably resulted in the significant mass transfer resistance since the large particles have a prolonged internal diffusion pathway, thus providing barrier for the penetration of the adsorbate deeper into the adsorbent structure^53^. In contrast, HD cages are readily accessible to l-Trp, so that there is no side effect of internal diffusion in HD because it is the zero-dimensional structures highly dispersed in the THTP_Cl.

### Generality of MOC-based Type II porous ionic liquid

We successfully extended the synthetic protocol to several representative MOCs. Specifically, six vanadium-based anionic MOCs, three zirconium-based cationic MOCs and two copper-based neutral MOCs were selected as pore hosts for the synthesis of various porous liquids under the same procedures. All selected MOCs are typical because they follow one of the three design principles including vertex-directed, face-directed and edge directed self-assembly, which afford MOCs with windows and faces, faces only or windows only, respectively. The details of all selected MOCs were listed in Supplementary Table 5. It is interesting to find that all charged MOCs can be readily dissolved in THTP_Cl, whereas both copper-based neutral MOCs exhibit poor solubility in THTP_Cl. The maximum solubility of each MOC in THTP were listed in Supplementary Table 5. The presence of MOCs after the dissolution into THTP_Cl were verified by QTOF-MS test for charged MOCs and MALDI-TOF test for neutral MOCs (Supplementary Fig. 26). The permanent porosities of all porous liquids were verified by the high-pressure methane uptakes which are all larger than THTP_Cl due to the occupation of methane molecules in MOCs. The uptake difference among all these MOC-based porous liquids are related to the concentration of MOCs in THTP_Cl and the pore size/volume of MOCs (Supplementary Fig. 27). As presented in Supplementary Table 5, all porous liquids exhibit observable fluidity, and the color of porous liquid is derived from the MOCs.

HD/THTP_Cl has been demonstrated to selectively extract l-Trp from aromatic amino acids mixture. We envisioned that HD/THTP_Cl can be extended to target the selectivity toward other biomolecules by utilizing the intrinsic cavity in HD/THTP_Cl. d-Ribose has been produced by microbial fermentation of d-glucose which are abundant in lignocellulosic biomass. The isolation and purification of d-ribose form fermentation broth is an important step during the whole fermentation process. In a typical experiment, the aqueous solution of equimolar d-ribose over d-glucose (2.5 mmol/L, 2 mL) was contacted with HD/THTP_Cl (0.1 mL). The mixture was stirred vigorously for 8 h at room temperature. The d-ribose uptake in HD/THTP_Cl (38.2 μmol/g) was much higher than pure THTP_Cl (5.5 μmol/g), and the number of d-ribose loaded per HD cage unit was calculated as 1.8. In contrast, the d-glucose uptake in pure THTP_Cl (5.3 μmol/g) was very close to HD/THTP_Cl (5.9 μmol/g), indicating the infeasible encapsulation of d-glucose in the HD cavity. These results suggest that d-ribose can be selectively extracted from the aqueous solution of d-ribose and d-glucose by HD/THTP_Cl. HD/THTP_Cl thereby has the potential to serve as the porous extraction media to isolate d-ribose in the preparative course.

In summary, HD/THTP_Cl is a Type II porous ionic liquid based upon coordination cages that induces a non-gaseous guest molecules selectivity. The modeling and several experimental studies provide structural insight into the existence of the permanent porosity in THTP_Cl with 6% HD. Compare with pure THTP_Cl, we have demonstrated how the empty cavity of HD, coupled with favorable host-guest interactions provided by the internal chemical environment of HD cage, affords HD/THTP_Cl with exceptional selectivity and recyclability in the context of industrially relevant l-Trp separation applications. Furthermore, the unit occupation by l-Trp in HD/THTP_Cl is much higher than other benchmark solid state porous material thanks to the pore size and dissolution of HD that enhance the diffusion of the l-Trp molecules into the intrinsic pores of HD/THTP_Cl. Perhaps most importantly, HD belongs to one of the most extensively studied and broadest classes of polyoxovanadate-based coordination cages^54^ and is therefore likely to serve as prototypal to promote a platform of related porous liquid with potential to resolve industrial challenges related to molecular separation.

## Methods

### Synthesis of DMA_4__HD

According to the previously reported procedure^34^, a mixture of 5-bromo-1,3-benzenedicarboxylatic acid (12.0 mg), VCl_3_ (20.0 mg) and 5 mL mixture solvent (DMF: H_2_O = 10:1) were put in a 20 mL scintillation vials and heated to 105 °C for 3 days until dry. The resulting residue were dissolved in DMF and dark green block crystals were obtained by slow diffusion of diethyl ether. Crystals were washed with menthol to give pure DMA_4__HD of 16.3 mg (yield of 46% based on the ligand). Elemental analysis calculated: C 27.38%; H 2.87%; N 4.47%; Experimental: C 26.48%; H 2.94%; N 4.53%. IR (KBr, cm^−1^): 3448 (br), 2783 (w), 1719 (m), 1603 (m), 1543 (s), 440 (s), 1409 (w), 1380 (vs), 1306 (w), 1288 (w), 974 (s), 733 (s), 721 (vs), 661 (w).

### Synthesis of HD/THTP_Cl

The hybrid nanodoughnut (DMA_4__HD, 0.36 g, 0.1 mmol, 1.0 eq.) was added in trihexyltetradecylphosphonium chloride (THTP_Cl, 5.19 g, 10 mmol, 100.0 eq.). The obtained mixtures were vigorously stirred at room temperature. The resulting green solution was added 5 mL acetonitrile and centrifuged to remove the precipitation. Finally, a green liquid was collected after rotary evaporation to remove acetonitrile.

### Structural analysis

Powder x-ray diffraction (PXRD) patterns of the samples were collected at 40 kV and 40 mA on a Rigaku SmartLab diffractometer with Cu Kα (λ = 1.5416 Å) radiation with 10°/min scan speed of and 0.02° in 2θ step size. ^1^H NMR spectrum was obtained on a Bruker 400 MHz spectrometer using D_2_O as solvent. Mass spectrum was obtained using an Agilent 6530 Q-TOF mass spectrometer in positive-ion detection mode. Dynamic Light Scattering (DLS) measurements were performed using a Zetasizer Nano ZS90 under 2 min thermal equilibrium. Elemental analysis was performed on elementar vario EL cube. High-pressure CH_4_ absorption data were collected using a gravimetric sorption analyzers-isoSORP^®^, from Rubotherm-TA instrument at 25 °C and a pressure range of 0–65 bar. MALDI-TOF mass spectrum were recorded on a Bruker UltrafleXtreme using DCTB as a matrix.

### High-pressure methane absorption

The methane uptakes were collected in a magnetic suspension balance (isoSORP from Rubotherm-TA^®^) at 25 °C and a pressure range of 0–65 bar. The absorption chamber was loaded with ca. 0.5 g of liquid sample residing the sample bucket. The sample was then evacuated at 50 °C under vacuum to remove any traces of dissolved gases until the weight remained constant. The chamber was then pressurized with dry methane at 25 °C, and the weight change was traced by a magnetic suspension balance. Each step with different pressures took around 40 mins before equilibrium.

### Thermal analysis

Thermal gravimetric analysis (TGA) was performed on Mettler Toledo TGA/DSC from 25 °C to 650 °C at 10 °C/min under N_2_ with a flow rate of 40 mL/min. Differential scanning calorimetry (DSC) measurements were taken under N_2_ atmosphere in the temperature range from −85 to 80 °C at a heating rate of 10 °C/min by using the DSC Q-20 TA instrument. To remove the thermal history, samples were precooled from 25 to −85 °C, then reheated from −85 to 80 °C to collect data.

### Rheologic analysis

Rheological properties were studied using Anton Paar MCR302. Strain-dependent and frequency-dependent rheology measurements were test by equipping CP25-2-SN27479 with a gap of 0.104 mm at room temperature. For temperature-dependent viscosity and stress versus shear rate measurements were test by equipping PP25-SN27504 with a gap of 0.100 mm under rotation mode.

### Extraction experiments

Typically, 0.10 mL of HD/THTP_Cl was mixed with 2.0 mL of l-Trp aqueous solution (0.05 mol/L) followed by vigorous stirring for 8 h at room temperature. The amino acids uptake were determined using a Agilent 1260 Infinity II Prime LC system equipped with G1311 Quatpump, G1314F wavelength detector and Agilent ZORBAX SB-C18 column (150 mm × 4.6 mm i.d., 5 μm particles). Amino acids were detected at specific wavelengths: 258 nm for l-Phe, 280 nm for l-Trp and l-Tyr. The mobile phase was composed of potassium dihydrogen phosphate aqueous solution (8 mmol/L) and HPLC-grade methanol with volumetric ratio of 9:1. The flow rate was set at 0.5 mL/min. The saccharides uptake were determined using a Agilent 1260 Infinity II Prime LC system equipped with an Agilent ZORBAX NH_2_ column (150 × 4.6 mm i.d., 5 μm particles) and a refractive index detector. The acetonitrile/water mixture (80/20, v/v) was used as the eluent. Calibration curve of amino acid were presented in Supplementary Figures. Extraction experiments were carried out in triplicate.

The Amino acid separation factor *S*_*i/j*_ was calculated according to Eq. (1):

1 $${S}_{i/j}=\left({x}_{i}/{x}_{j}\right)/\left({y}_{i}/{y}_{j}\right)$$

where *x* is the mole fraction in HD/THTP_Cl phase, *y* is the mole fraction in the aqueous phase at equilibrium, *i* represents l-Trp, and *j* represents any other amino acid, respectively.

Structure unit occupation was calculated according to Eq. (2):

2 $${{{{{{\rm{Structure}}}}}}\; {{{{{\rm{unit}}}}}}\; {{{{{\rm{occupation}}}}}}}={q}_{e}\times M$$

where *q*_e_ is the gravimetric uptake of amino acids that are immobilized by either HD cage or MOF structure (mol/g), *M* is the molecular weight of one HD cage or the molecular weight contained in one unit cell of MOF structure (g/mol). *M*_HD_ = 3412.9 g/mol, *M*_MIL-140B_ = 2571.2 g/mol, *M*_MIL-140C_ = 2779.5 g/mol, *M*_MIL-140D_ = 3554.7 g/mol, *M*_MIL-68(Al)_ =2497.3 g/mol, *M*_UIO-66_ =1726.4 g/mol, *M*_MOF-808_ =1387.5 g/mol, *M*_HY_ = 11530.8 g/mol)

### Computational details

All the all-atom MD simulations were based on a gromos54a7 force field^55^ by Automated Topology Builder (ATB)^56^ and were carried out using the Gromacs-4.6.7 software package^57^. The system is a relaxed liquid configuration at 298 K. The total run time was 10 ns NPT for the equilibrium MD simulation. We used the relaxed system as a starting configuration. As it is prior to system relaxation MD, energy minimization was carried out with a composite protocol of steepest descent using termination gradients of 100 kJ/mol·nm. The Nose´-Hoover thermostat^58^ was used to maintain the equilibrium temperature at 298 K and periodic boundary conditions were imposed on all three dimensions. The Particle Mesh-Ewald method^59,60^ was used to compute long-range electrostatics within a relative tolerance of 1 × 10^−6^. A cut-off distance of 1 nm was applied to real-space Ewald interactions. The same value was used for van der Waals interactions. The LINCS algorithm^61^ was applied to constrain bond lengths of hydrogen atoms. A leap-frog algorithm^62^ was used with a time step of 2 fs. The wave function were generated with the GFN-xTB method^63^ using xtb 6.3 software. The structure of the input xtb software comes from the equilibrium state in the MD simulation. Calculation of reduced density gradient^50^ and second largest eigenvalue of the electron density hessian (λ_2_) were performed in Multiwfn 3.7 program^52^. The molecular structures can be visualized through visual molecular dynamics software (VMD, version 1.9.3)^64^. The binding energies were calculated by utilizing the g_mmpbsa^65^ of GROMACS.

## Supplementary information

Supplementary Information

## Data Availability

All data that support the findings of this study are available within the paper and its Supplementary Information or from the corresponding author upon request. Source data are provided with this paper.

## Source data

Source Data
