# Supplementary material for: Type II porous ionic liquid based on metal-organic cages that enables l-tryptophan identification
Source: Nat Commun. 2022 Apr 29;13:2353. doi: 10.1038/s41467-022-30092-2 (PMC9054828; doi:10.1038/s41467-022-30092-2)
Supplement: Supplementary file 1 — Supplementary Information [file 41467_2022_30092_MOESM1_ESM.pdf]

Supplementary Information

Type II porous ionic liquid based on metal-organic cage that enables L-tryptophan identification

Zhuxiu Zhang[1], Baolin Yang[1], Bingjie Zhang[1], Mifen Cui[1], Jihai Tang*[1,2], Xu Qiao*[1,2]

[1]State Key Laboratory of Materials-Oriented Chemical Engineering,
College of Chemical Engineering, Nanjing Tech University, No. 30 Puzhunan Road, Nanjing 211816, China.
[2]Jiangsu National Synergetic Innovation Centre for Advanced Materials (SICAM), No. 5 Xinmofan Road, Nanjing 210009, China.

E-mail: jhtang@njtech.edu.cn, qct@njtech.edu.cn

Table of contents:

Supplementary Fig. 1 ^{1}H NMR (D_2O, 400 MHz, 298 K) of the white precipitate corresponding to the formation of dimethylammonium chloride formed upon addition of acetonitrile to DMA_4_HD/THTP_Cl. 1

Supplementary Fig. 2 TGA curves of HD/THTP_Cl and THTP_Cl. 1

Supplementary Fig. 3 Stress versus shear rate plots of HD/THTP_Cl measured at room temperature under rotation mode 2

Supplementary Fig. 4 Viscosity versus shear rate plots of HD/THTP_Cl measured at room temperature under rotation mode 2

Supplementary Fig. 5 The number of occupied methane molecules in the HD cage as a function of time during the production run at 25 °C and 1 bar (a), 5 bar (b), 10 bar (c) and 65 bar (d). 3

Supplementary Fig. 6 L-Phe uptake over time in HD/THTP_Cl and THTP_Cl 4

Supplementary Fig. 7 L-Tyr uptake over time in HD/THTP_Cl and THTP_Cl. 4

Supplementary Fig. 8 RMSD for L-Trp simulation system. 5

Supplementary Fig. 9 RMSD for L-Phe simulation system. 5

Supplementary Fig. 10 RMSD for L-Tyr simulation system. 6

Supplementary Fig. 11 RDG color bar 6

Supplementary Fig. 12 The van der Walls interactions between the encapsulated-L-Trp and HD cage by the RDG method. 7

Supplementary Fig. 13 The van der Walls interactions between the encapsulated-L-Trp and HD cage by the RDG method. 7

Supplementary Fig. 14 The C-H···π interaction between CH_2 group and the benzene ring of ligand. 8

Supplementary Fig. 15 The N-H···O hydrogen bond between between the nitrogen atom in the indole ring of L-Trp and terminal oxygen atom in the polyoxovanadate. 8

Supplementary Fig. 16 The weak N-H···O hydrogen bond between between the nitrogen atom in the indole ring of L-Trp and terminal oxygen atom from HD cage. 8

Supplementary Fig. 17 The weak N-H···O hydrogen bond between between the nitrogen atom in the indole ring of L-Trp and terminal oxygen atom from HD cage. 9

Supplementary Fig. 18 The weak N-H···O hydrogen bond between between the nitrogen atom in the indole ring of L-Trp and bridge oxygen atom from HD cage. 9

Supplementary Fig. 19 The simulation box of L-Tyr simulation system. Snapshot of empty HD without L-Tyr molecule in its cavity.. 9

Supplementary Fig. 20 The N-H···O interaction between the nitrogen atom in the amine group of L-Tyr and terminal oxygen atom from HD cage. 10

Supplementary Fig. 21 The N-H···O interaction between the nitrogen atom in the amine group of L-Tyr and terminal oxygen atom from HD cage. 10

Supplementary Fig. 22 The simulation box of L-Phe simulation system. Snapshot of empty HD without L-Phe molecule in its cavity. 10

Supplementary Fig. 23 The distance between the nitrogen atom the amine group of L-Phe and terminal oxygen atom from HD cage. 11

Supplementary Fig. 24 Center-of-mass – center-of-mass PMF for HD – amino acid molecule from MD simulations. 11

Supplementary Fig. 25 Structure unit occupation of porous materials against structure unit volume and L-Trp uptakes. 11

Supplementary Fig. 26 Mass spectrum of various porous liquids based upon anionic, cationic and neutral MOCs.. 12

Supplementary Fig. 27 High pressure CH_4 absorption data in THTP_Cl and MOC-based porous liquids at 25 °C. 13

Supplementary Fig. 28 Calibration curve for the determination of L-Trp (a), L-Phe (b), L-Tyr (c), D-ribose (d) and D-glucose (e) 13

Supplementary Table 1 The average number of methane molecules occupied in HD cages. 14

Supplementary Table 2 RMSD values obtained from the MD simulations. 14

Supplementary Table 3 The amino acid uptake on other porous liquid. 14

Supplementary Table 4 Binding energy of HD/THTP_Cl and amino acids......................... 14

Supplementary Table 5 The synthesis of various porous liquids based upon anionic, cationic and neutral MOCs. 15

Supplementary references 18

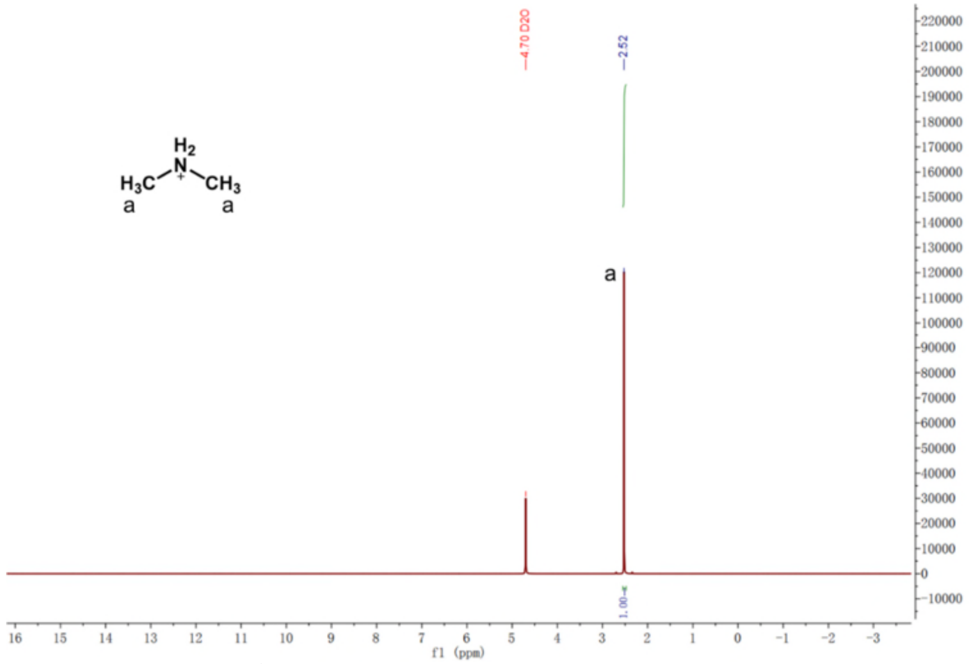

Supplementary Fig. 1 ^{1}H NMR (D_2O, 400 MHz, 298 K) of the white precipitate corresponding to the formation of dimethylammonium chloride formed upon addition of acetonitrile to DMA_4_HD/THTP_Cl.

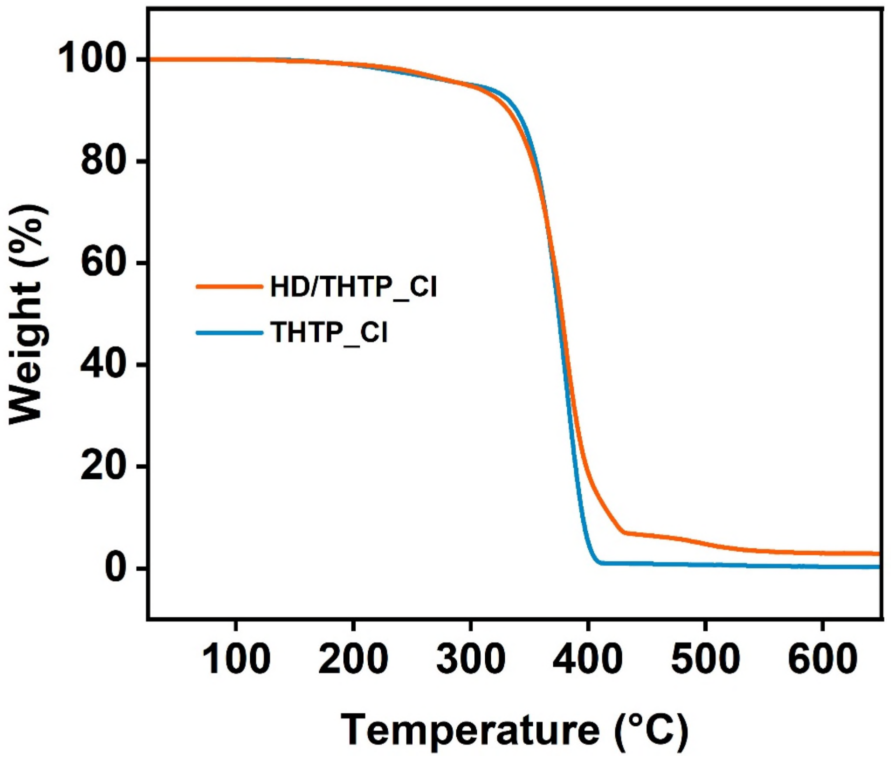

Supplementary Fig. 2 TGA curves of HD/THTP_Cl and THTP_Cl.

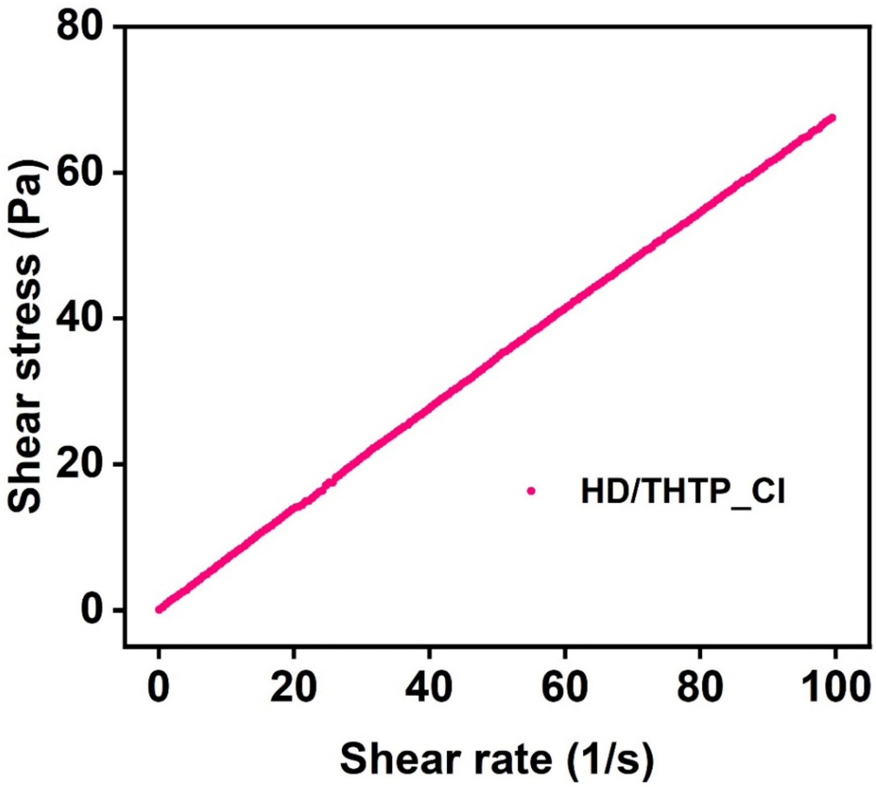

Supplementary Fig. 3 Stress versus shear rate plots of HD/THTP_Cl measured at room temperature under rotation mode.

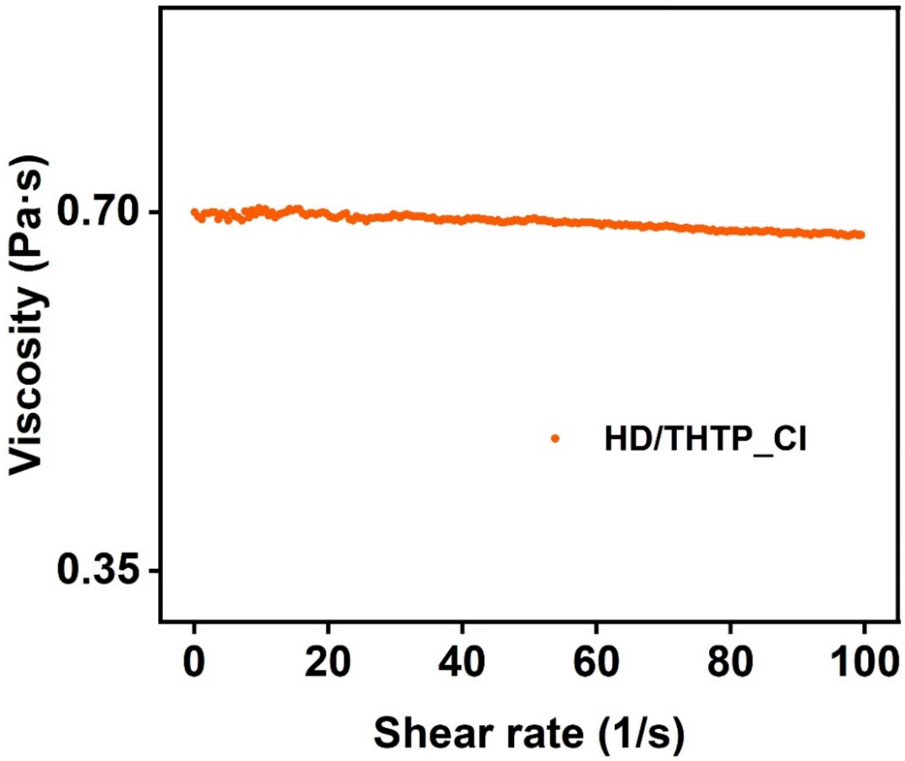

Supplementary Fig. 4 Viscosity versus shear rate plots of HD/THTP_Cl measured at room temperature under rotation mode.

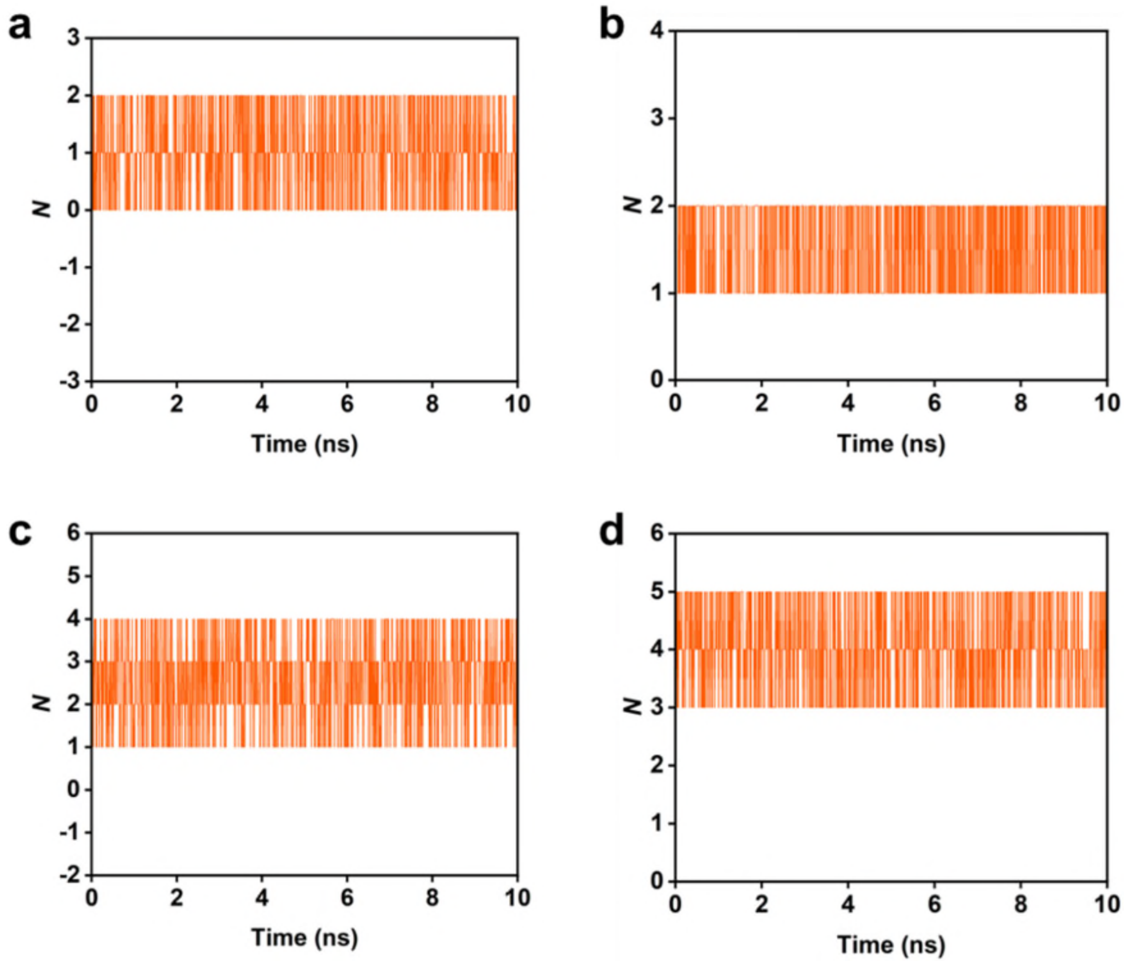

Supplementary Fig. 5 The number of occupied methane molecules in the HD cage as a function of time during the production run at 25 °C and 1 bar (a), 5 bar (b), 10 bar (c) and 65 bar (d).

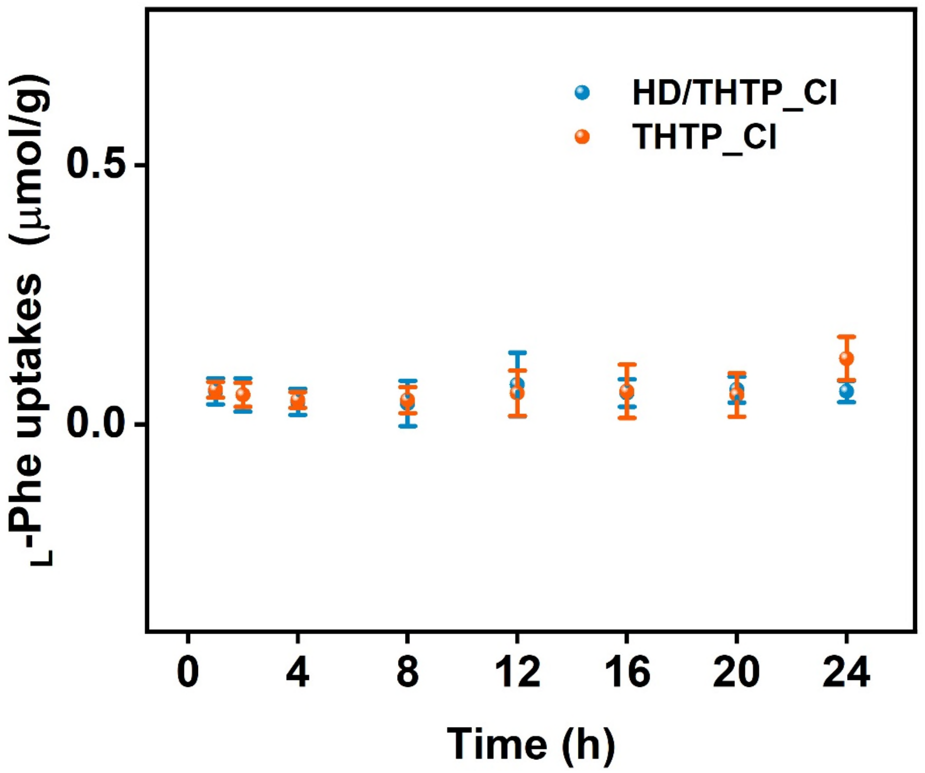

Supplementary Fig. 6 L-Phe uptake over time in HD/THTP_Cl and THTP_Cl.

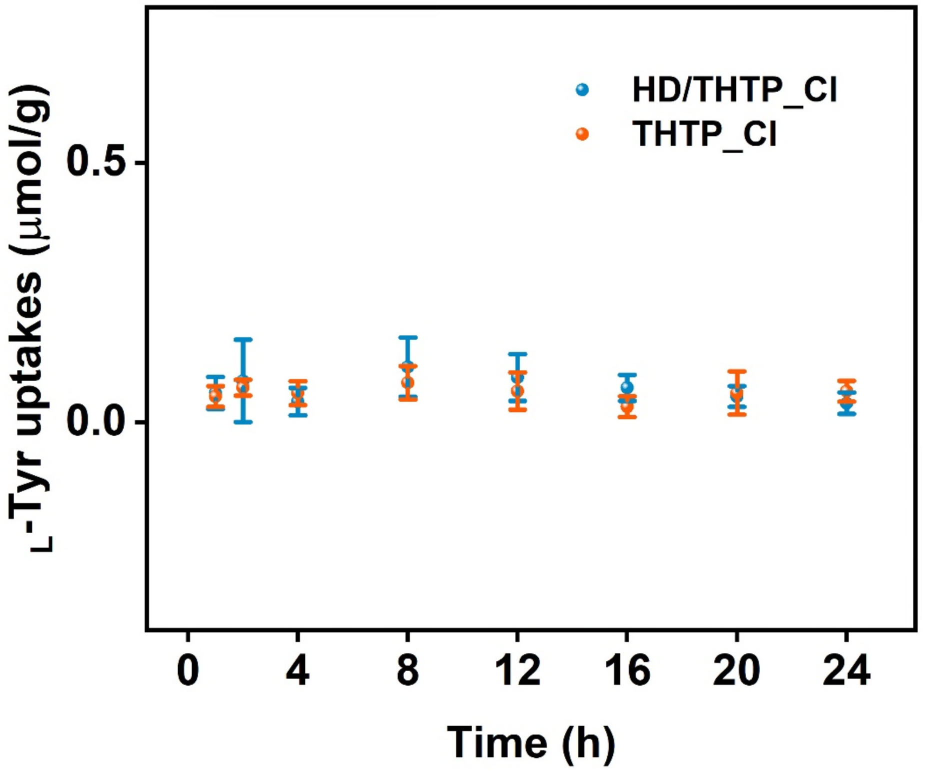

Supplementary Fig. 7 L-Tyr uptake over time in HD/THTP_Cl and THTP_Cl.

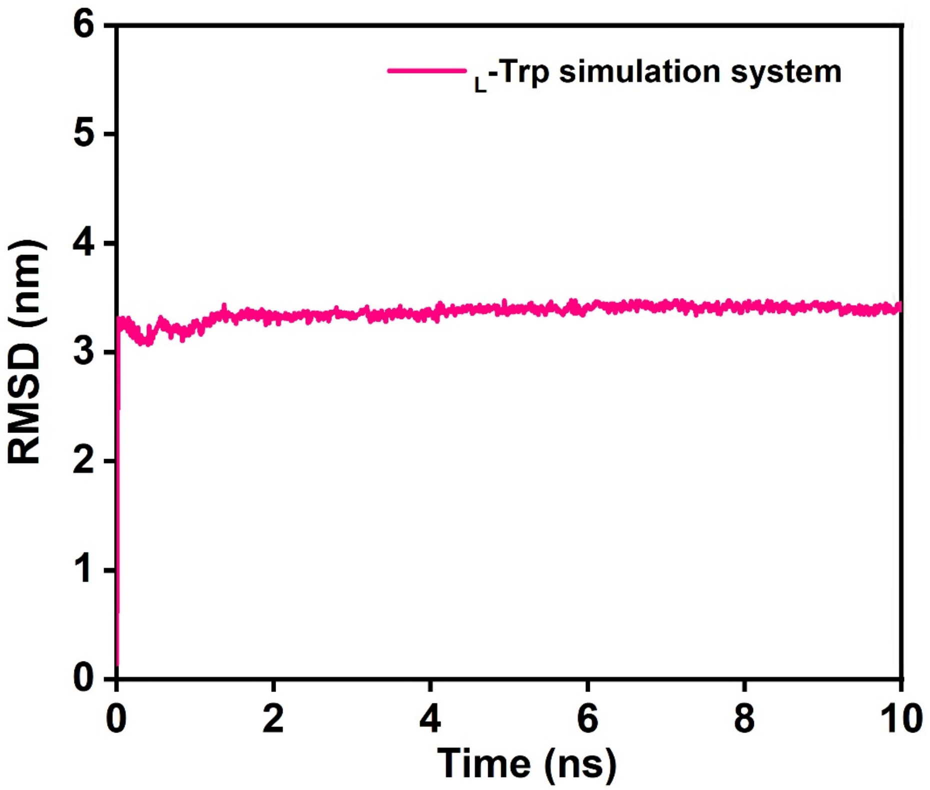

Supplementary Fig. 8 RMSD for L-Trp simulation system.

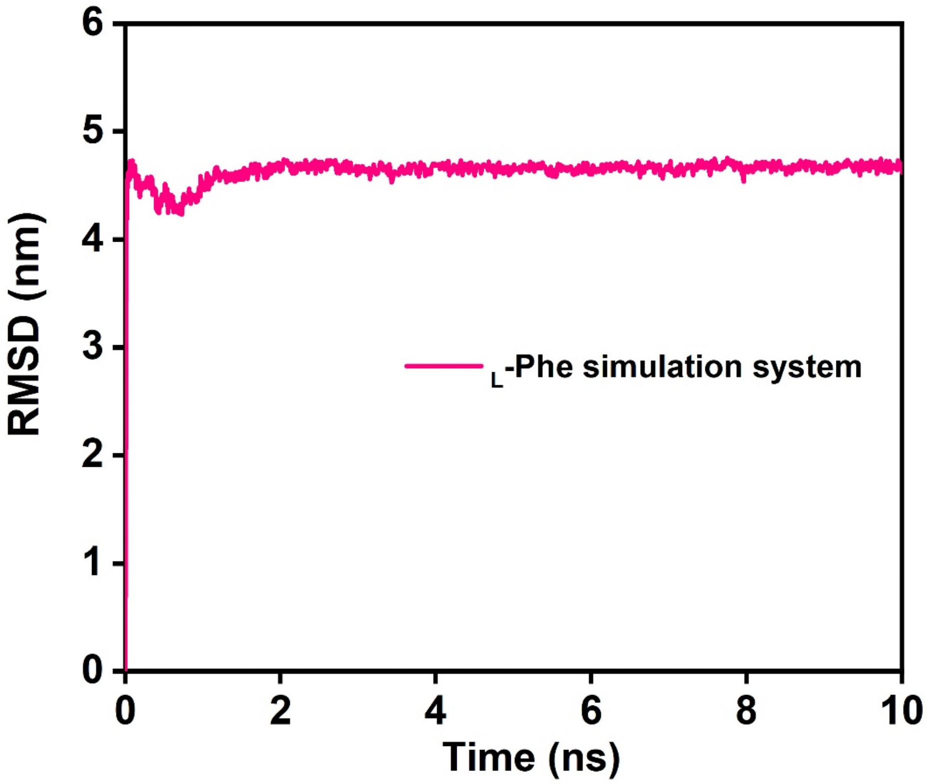

Supplementary Fig. 9 RMSD for L-Phe simulation system.

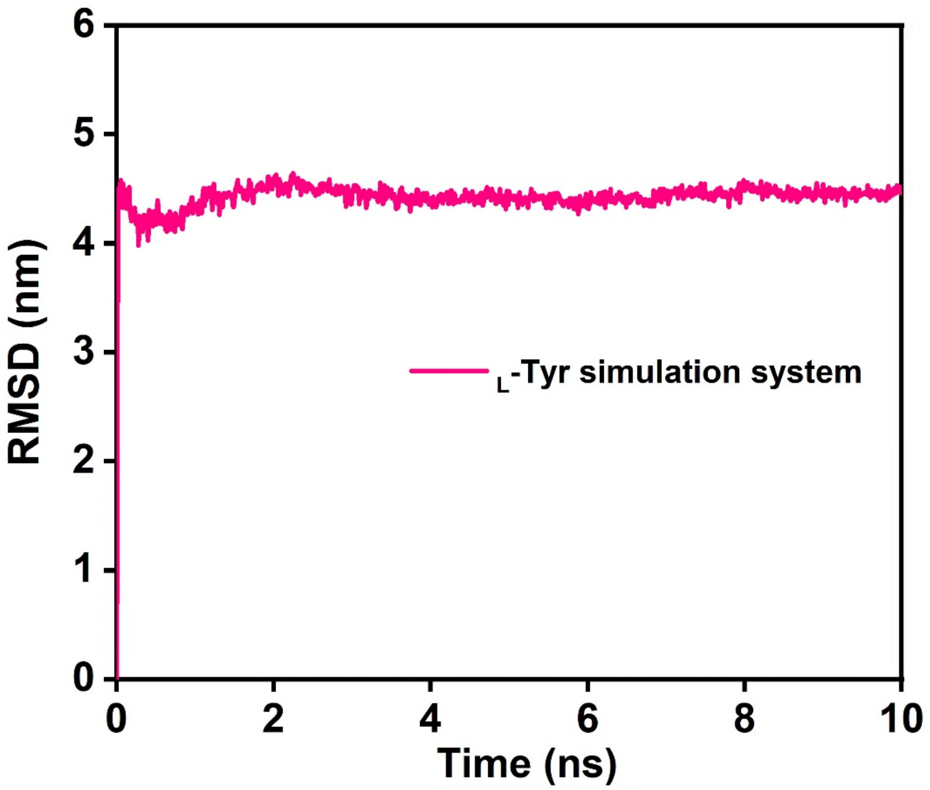

Supplementary Fig. 10 RMSD for L-Tyr simulation system.

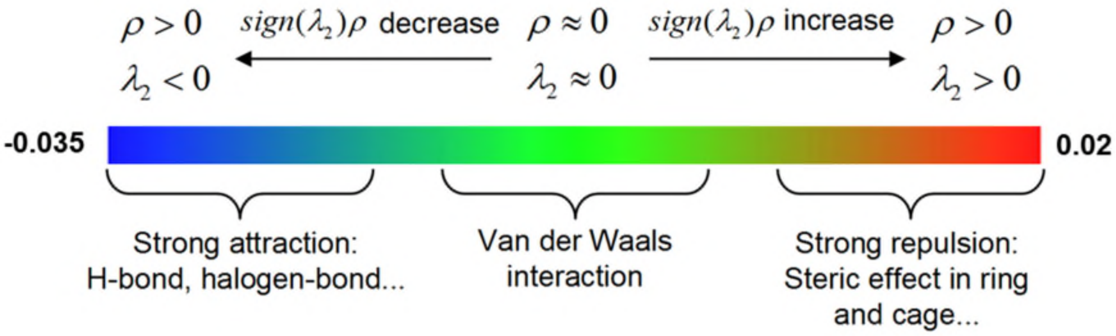

Supplementary Fig. 11 RDG color bar.

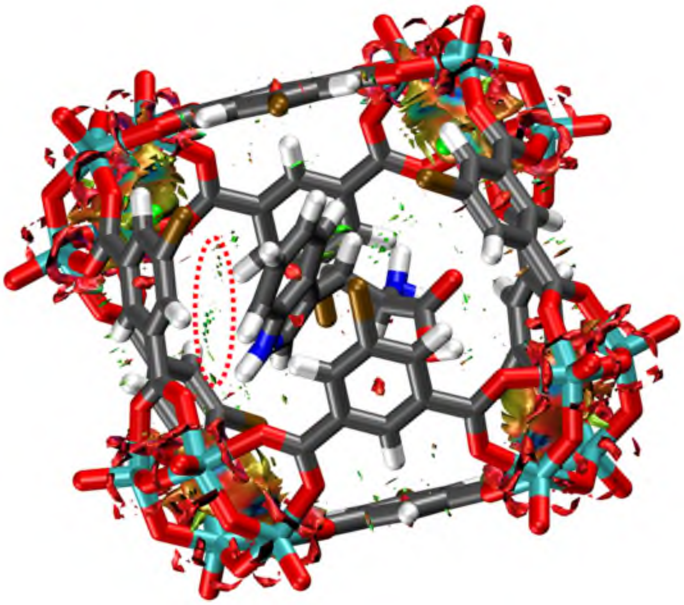

Supplementary Fig. 12 The van der Walls interactions between the encapsulated-L-Trp and HD cage by the RDG method.

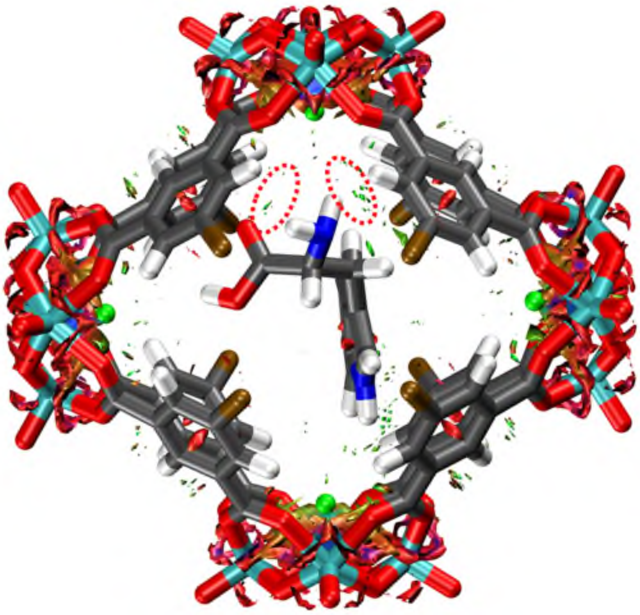

Supplementary Fig. 13 The van der Walls interactions between the encapsulated-L-Trp and HD cage by the RDG method.

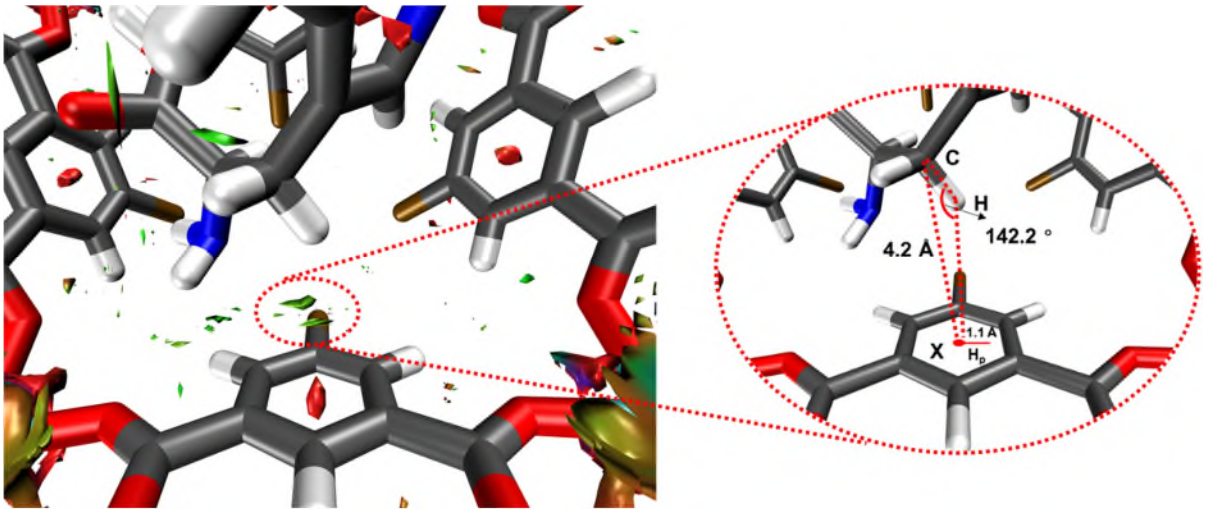

Supplementary Fig. 14 The C-H···π interaction between CH_2 group and the benzene ring of ligand. Geometric parameters are measured according to the Brandl-Weiss system for defining C-H···π interaction: (1) the distance between the donor carbon and the ring centroid of the aromatic system ($d_{C–X} \leq 4.5$ Å), (2) the angle formed by the donor carbon, hydrogen and ring centroid ($\angle C–H–X \geq 120°$), (3) the distance of the vertical projection of the donor H-atom onto the acceptor π system from the geometric center of the aromatic system ($d_{Hp–X}$ = 1–1.2 Å).

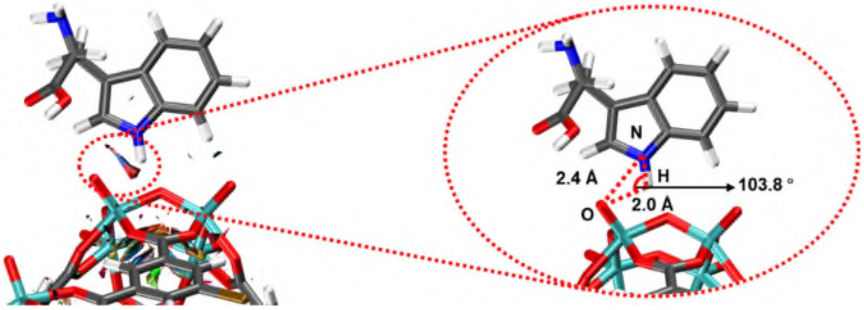

Supplementary Fig. 15 The N-H···O hydrogen bond between between the nitrogen atom in the indole ring of L-Trp and terminal oxygen atom in the polyoxovanadate.

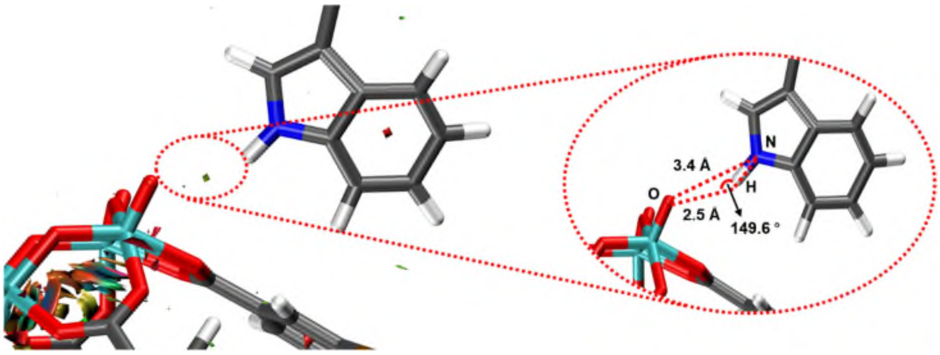

Supplementary Fig. 16 The weak N-H···O hydrogen bond between between the nitrogen atom in the indole ring of L-Trp and terminal oxygen atom from HD cage.

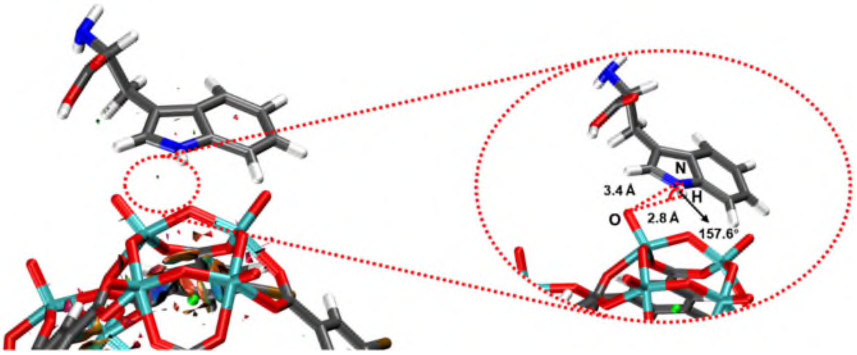

Supplementary Fig. 17 The weak N-H···O hydrogen bond between between the nitrogen atom in the indole ring of L-Trp and terminal oxygen atom from HD cage.

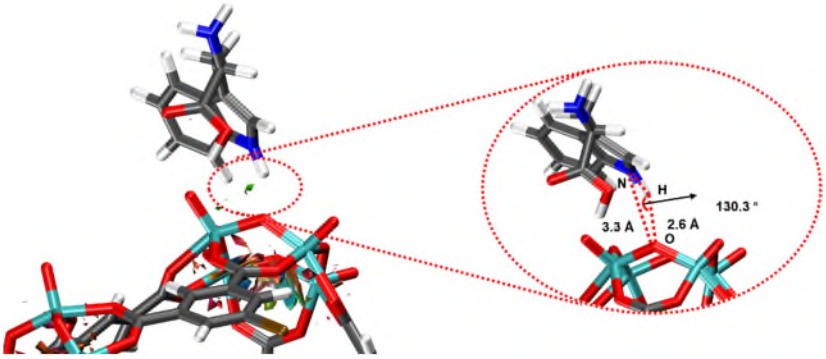

Supplementary Fig. 18 The weak N-H···O hydrogen bond between between the nitrogen atom in the indole ring of L-Trp and bridge oxygen atom from HD cage.

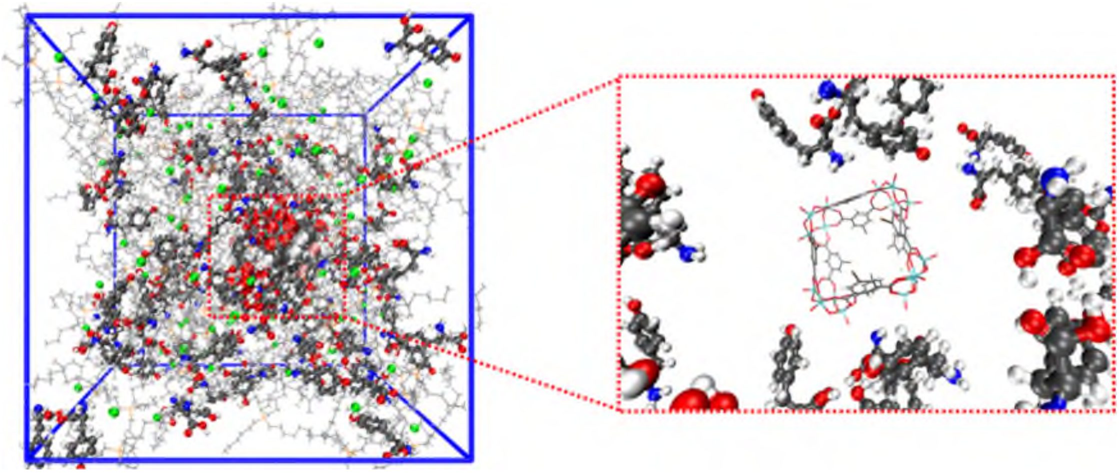

Supplementary Fig. 19 The simulation box of L-Tyr simulation system. Snapshot of empty HD without L-Tyr molecule in its cavity.

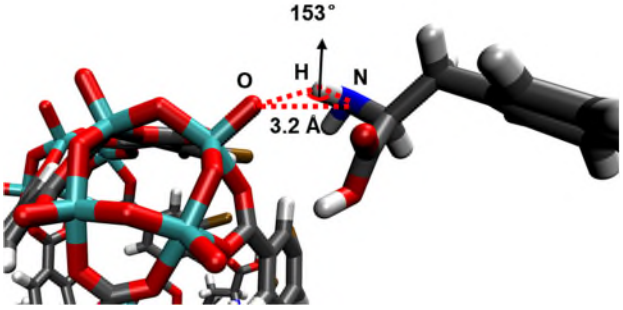

Supplementary Fig. 20 The N-H···O interaction between the nitrogen atom in the amine group of L-Tyr and terminal oxygen atom from HD cage.

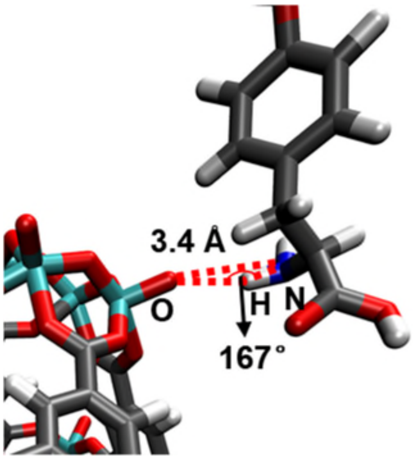

Supplementary Fig. 21 The N-H···O interaction between the nitrogen atom in the amine group of L-Tyr and terminal oxygen atom from HD cage.

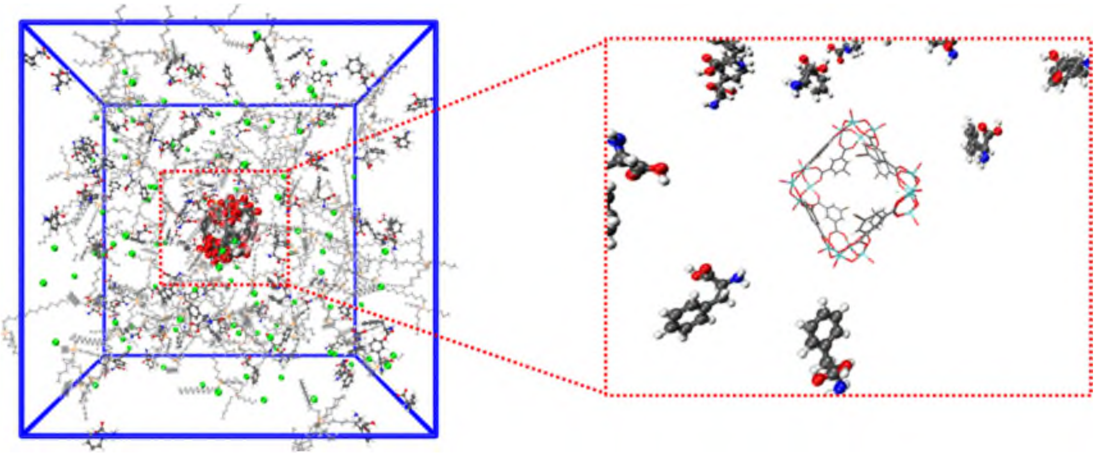

Supplementary Fig. 22 The simulation box of L-Phe simulation system. Snapshot of empty HD without L-Phe molecule in its cavity.

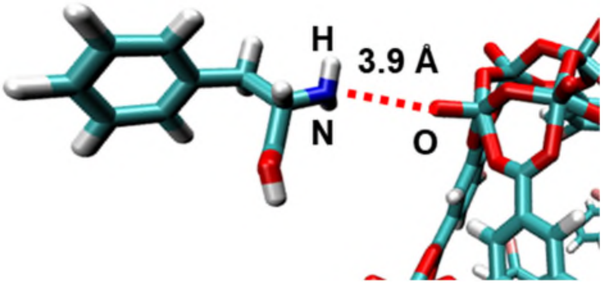

Supplementary Fig. 23 The distance between the nitrogen atom the amine group of L-Phe and terminal oxygen atom from HD cage.

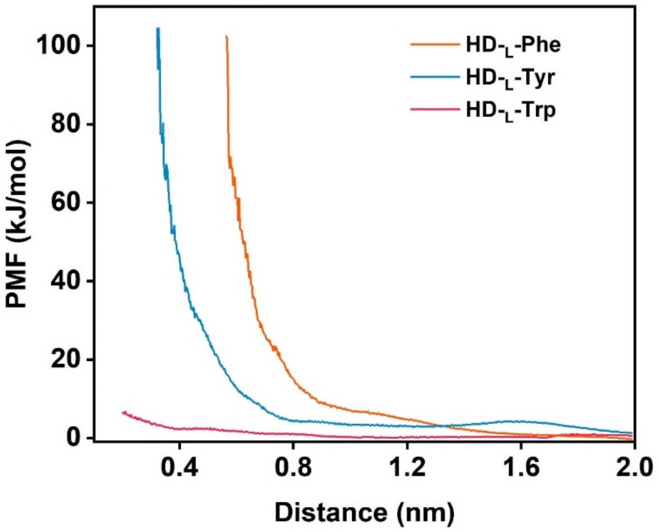

Supplementary Fig. 24 Center-of-mass – center-of-mass PMF for HD – amino acid molecule from MD simulations.

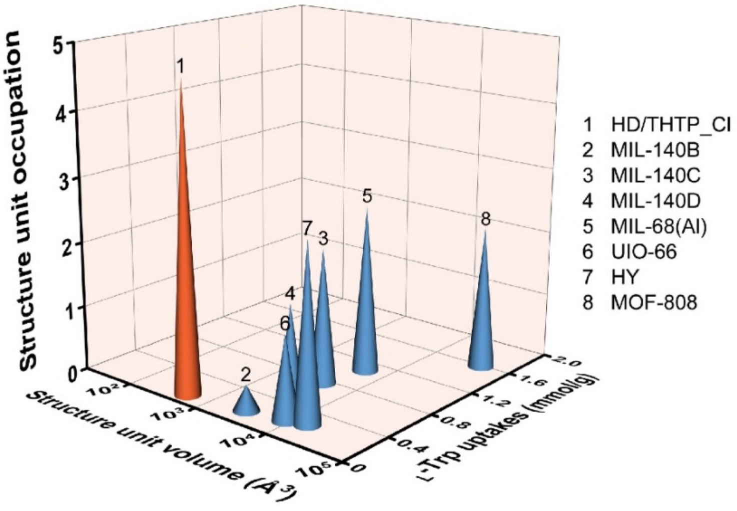

Supplementary Fig. 25 Structure unit occupation of porous materials against structure unit volume and L-Trp uptakes.

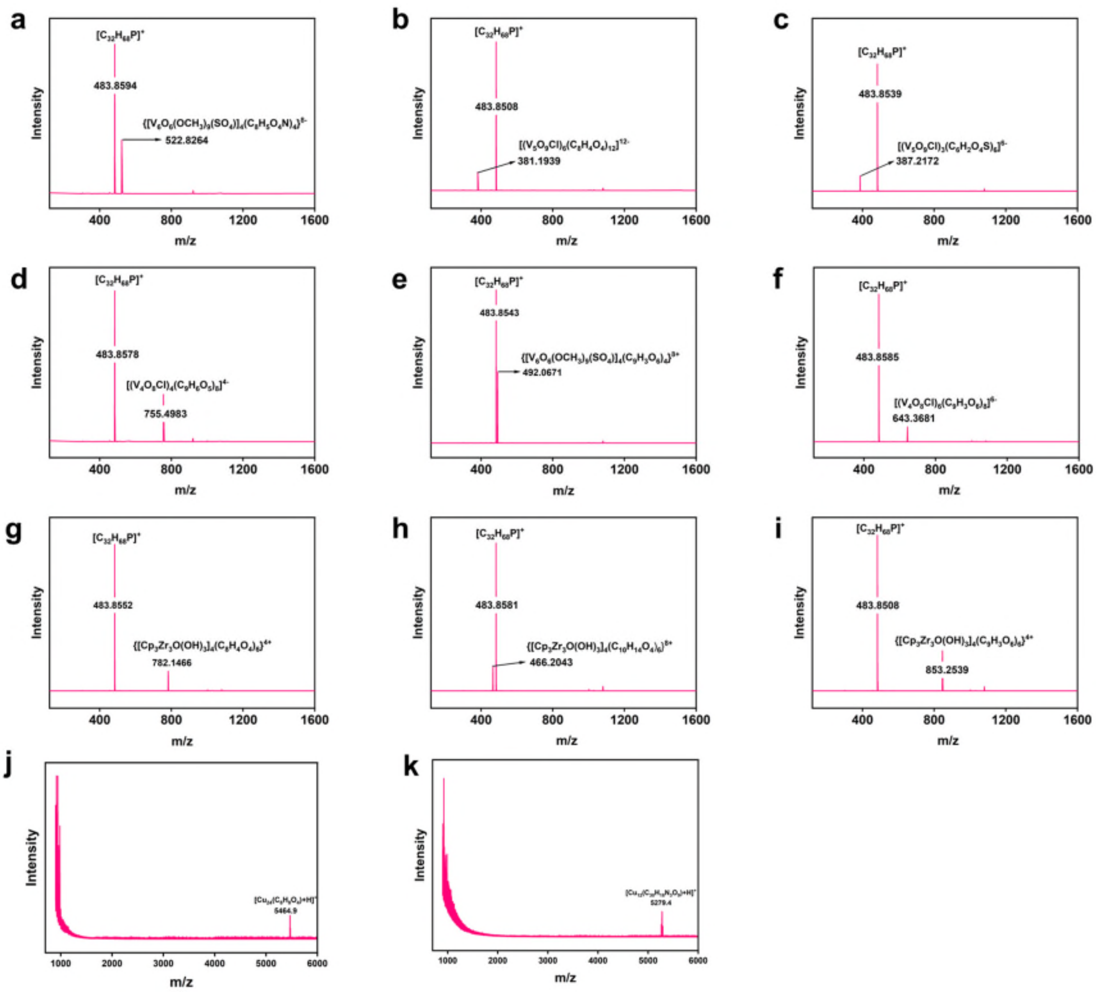

Supplementary Fig. 26 Mass spectrum of various porous liquids based upon anionic, cationic and neutral MOCs. QTOF mass spectrum of VMOP-12/THTP_Cl (a), VMOP-1/THTP_Cl (b), VMOP-4/THTP_Cl (c), HD-OCH_3/THTP_Cl (d), VMOP-14/THTP_Cl (e), Hyball-3/THTP_Cl (f), ZrT-1/THTP_Cl (g), HCCF-1/THTP_Cl (h) and ZrT-2/THTP_Cl (i). MALDI-TOF-MS spectrum of MOP-1/THTP_Cl (j) and MOP-Tr/THTP-Cl (k).

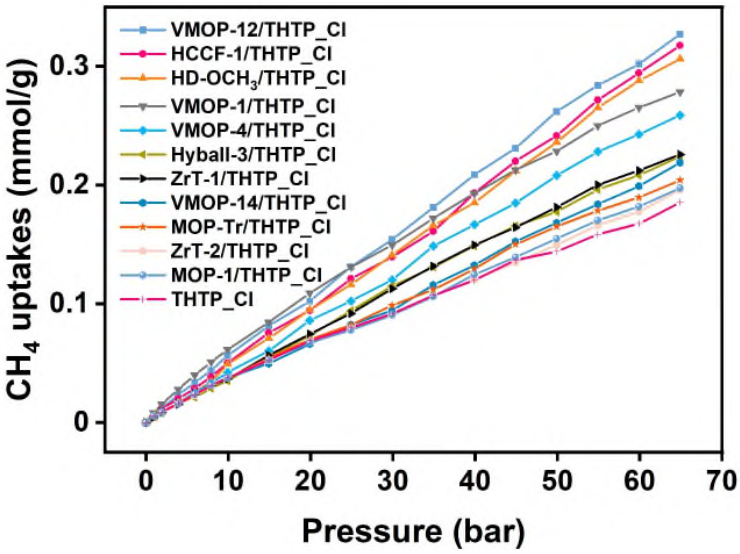

Supplementary Fig. 27 High pressure CH_4 absorption data in THTP_Cl and MOC-based porous liquids at 25 ℃.

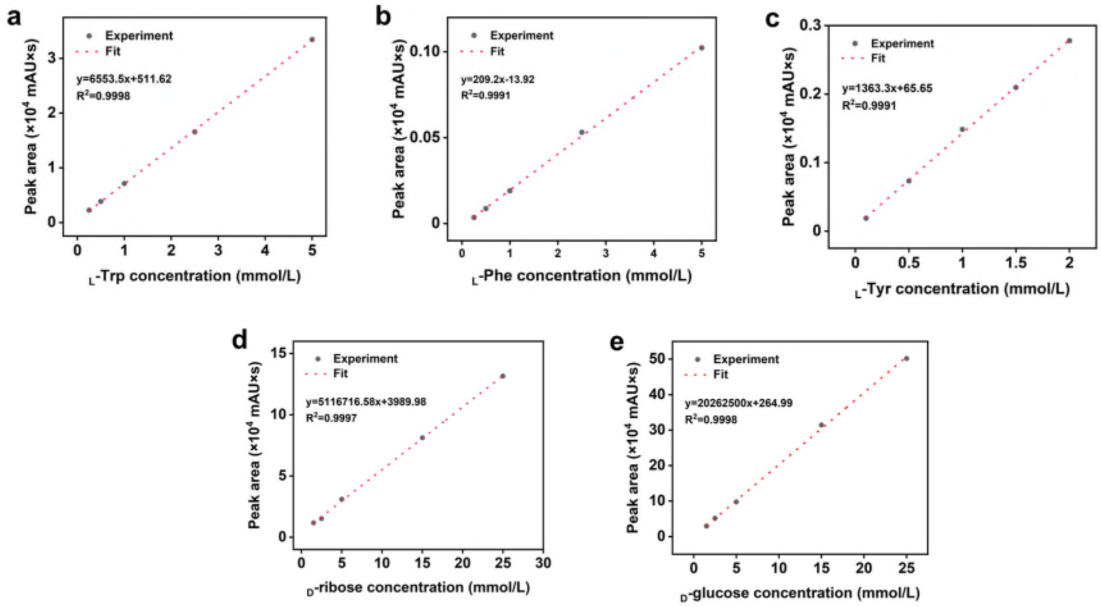

Supplementary Fig. 28 Calibration curve for the determination of L-Trp (a), L-Phe (b), L-Tyr (c), D-ribose (d) and D-glucose (e)

Supplementary Table 1 The average number of methane molecules occupied in HD cages.

Pressure（bar）	From MD simulation	From high pressure absorption data	Deviation between simulated and experimental value
1	0.99	0.30	0.697
5	1.51	0.72	0.523
10	2.56	1.25	0.512
65	4.72	5.18	0.097

Supplementary Table 2 RMSD values obtained from the MD simulations.

System	RMSD (nm)
L-Trp simulation system	3.38 ± 0.043
L-Phe simulation system	4.66 ± 0.035
L-Tyr simulation system	4.43 ± 0.050

Supplementary Table 3 The amino acid uptake on other porous liquid.

Type	Compound name	L-Trp uptake (μmol/g)	L-Phe uptake (μmol/g)	L-Tyr uptake (μmol/g)
II	MOP-18 dissolved in 15-crown-5[1] (MOP-18/15-Crown-5)	185.6 ± 0.3	348.6 ± 1.2	16.1 ± 0.2
	$CC3^3$:13^3-R dissolved in hexachloropropene[2] ($CC3^3$:13^3-R/HCP)	0.02 ± 0.003	0.05 ± 0.01	0.01 ± 0.006
III	H-ZSM-5 dispersed in trihexyltetradecylphosphonium bromide[3] (H-ZSM-5/THTP_Br)	28.1 ± 0.6	14.2 ± 0.5	45.8 ± 0.7
	ZIF-8 dispersed in trihexyltetradecylphosphonium chloride[4] (ZIF-8/THTP_Cl)	120.5 ± 1.5	0.9 ± 0.02	92.6 ± 0.6

Supplementary Table 4 Binding energy of HD/THTP_Cl and amino acids.

Compound	Binding Energy / kJ·mol $^{-1}$
HD-L-Trp	-121.23
HD-L-Phe	-91.42
HD-L-Tyr	-89.53
THTP-L-Trp	-102.45
THTP-L-Phe	-90.64
THTP-L-Tyr	-87.99

Supplementary Table 5 The synthesis of various porous liquids based upon anionic, cationic and neutral MOCs.

Vanadium-based anionic MOCs					
Design principle	Polyhedron type	Pore host	Crystal structure	Appearance	Solubility (mg/mL)
Edge-directed	Tetrahedron	VMOP-12[5]	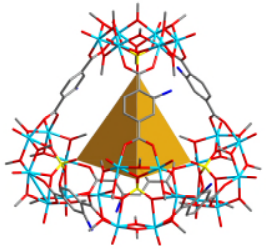	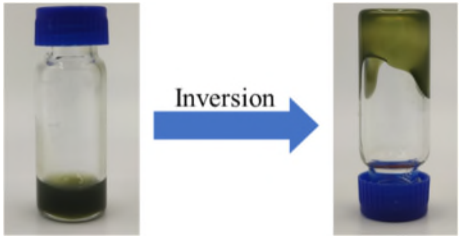	67.3
	Octahedron	VMOP-1[6]	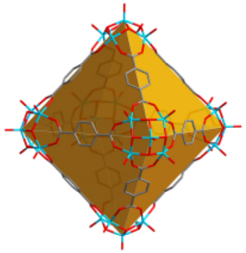	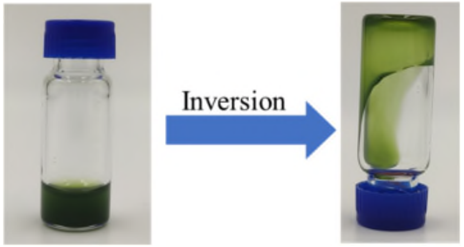	90.1
	Triangle	VMOP-4[7]	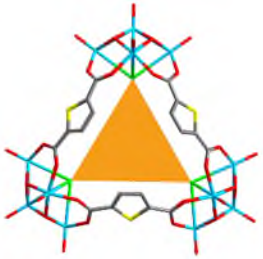	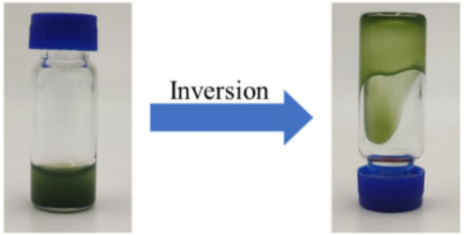	23.2
	Square	HD-OCH$_3$[8]	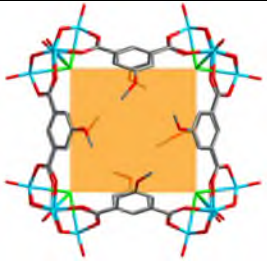	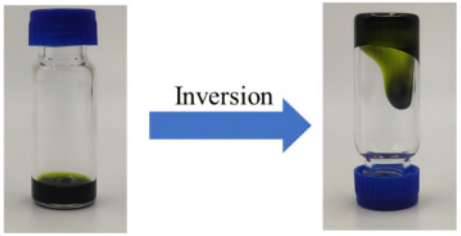	54.2

Face-directed	Tetrahedron	VMOP-14[5]	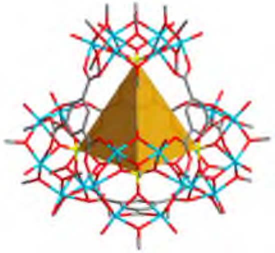	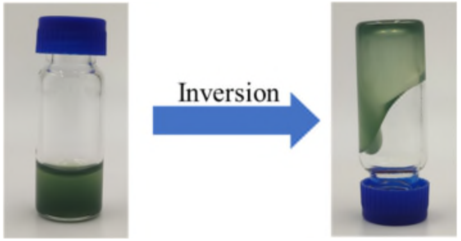	67.8
	Octahedron	Hyball-3[9]	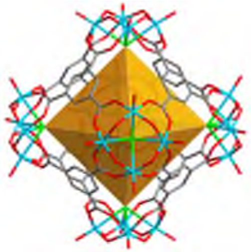	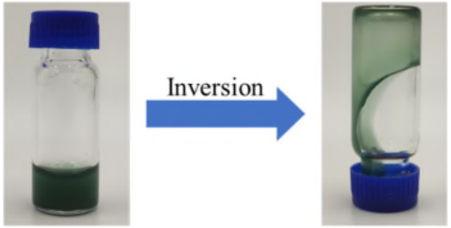	53.8
Zirconium-based cationic MOCs					
Design principle	Polyhedron	Pore host	Crystal structure	Appearance	Solubility (mg/mL)
Edge-directed	Tetrahedron	ZrT-1[10]	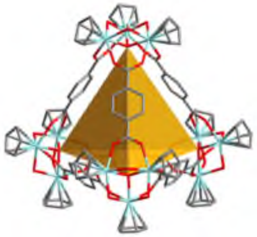	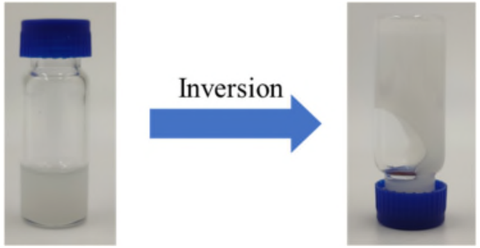	38.4
	Cube	HCCF-1[11]	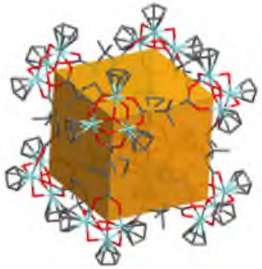	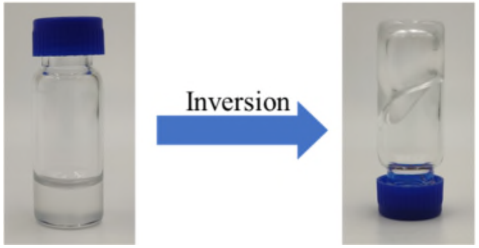	65.8

Face-directed	Tetrahedron	ZrT-2[10]	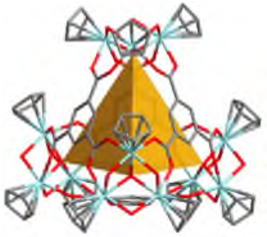	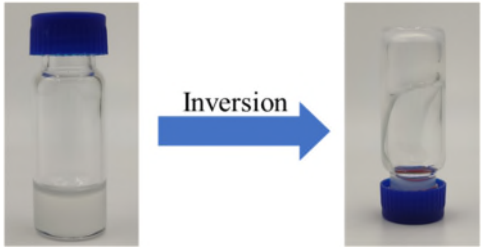	44.8
Copper-based neutral MOCs					
Design principle	Polyhedron	Pore host	Crystal structure	Appearance	Solubility (mg/mL)
Edge-directed	Cuboctahedron	MOP-1[12]	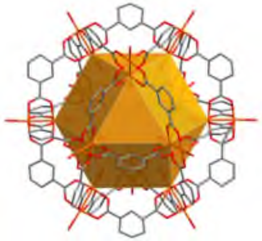	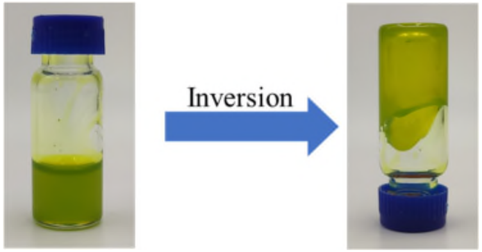	8.1
Face-directed	Octahedron	MOP-Tr[13]	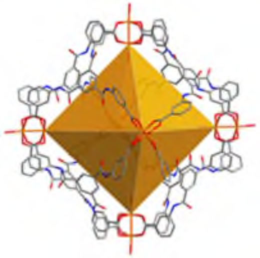	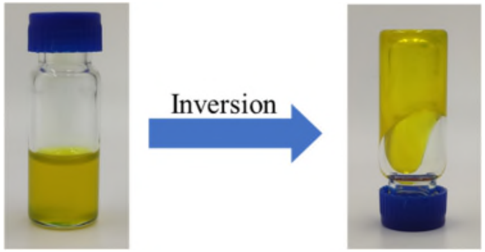	6.5

Supplementary references

1 Deng, Z. *et al.* Facilitate gas transport through metal–organic polyhedra constructed porous liquid membrane. *Small* **16**, 1907016, (2020).

2 Egleston, B. D. *et al.* Controlling gas selectivity in molecular porous liquids by tuning the cage window size. *Angewandte Chemie International Edition* **59**, 7362-7366, (2020).

3 Li, P. *et al.* Porous liquid zeolites: hydrogen bonding-stabilized H-ZSM-5 in branched ionic liquids. *Nanoscale* **11**, 1515-1519, (2019).

4 Zhou, Y. *et al.* Integrated, one-pot carbon capture and utilisation using porous ionic liquids. *Chemical Communications* **57**, 7922-7925, (2021).

5 Zhang, Y. *et al.* Anderson-like alkoxo-polyoxovanadate clusters serving as unprecedented second building units to construct metal–organic polyhedra. *Chemical Communications* **52**, 9632-9635, (2016).

6 Zhang, Y. *et al.* Polyoxovanadate-based organic–inorganic hybrids: from $\{V_5O_9Cl\}$ clusters to nanosized octahedral cages. *Dalton Transactions* **45**, 3698-3701, (2016).

7 Zhang, Y. *et al.* Ligand-directed assembly of polyoxovanadate-based metal–organic polyhedra. *Inorganic Chemistry* **55**, 8770-8775, (2016).

8 Zhang, Z., Gao, W., Wojtas, L., Zhang, Z. & Zaworotko, M. J. A new family of anionic organic-inorganic hybrid doughnut-like nanostructures. *Chemical Communications* **51**, 9223-9226, (2015).

9 Zhang, Z., Wojtas, L. & Zaworotko, M. J. Organic–inorganic hybrid polyhedra that can serve as supermolecular building blocks. *Chemical Science* **5**, 927-931, (2014).

10 Liu, G., Ju, Z., Yuan, D. & Hong, M. In situ construction of a coordination zirconocene tetrahedron. *Inorganic Chemistry* **52**, 13815-13817, (2013).

11 Ju, Z., Liu, G., Chen, Y.-S., Yuan, D. & Chen, B. From coordination cages to a stable crystalline porous hydrogen-bonded framework. *Chemistry – A European Journal* **23**, 4774-4777, (2017).

12 Moulton, B., Lu, J., Mondal, A. & Zaworotko, M. J. Nanoballs: nanoscale faceted polyhedra with large windows and cavities. *Chemical Communications*, 863-864, (2001).

13 Prakash, M. J. *et al.* Metal−Organic Polyhedron based on a Cu^{II} paddle-wheel secondary building unit at the truncated octahedron corners. *Inorganic Chemistry* **48**, 1281-1283, (2009).
